# Genome-wide identification and characterization of microRNAs by small RNA sequencing for low nitrogen stress in potato

**DOI:** 10.1371/journal.pone.0233076

**Published:** 2020-05-19

**Authors:** Jagesh Kumar Tiwari, Tanuja Buckseth, Rasna Zinta, Aastha Saraswati, Rajesh Kumar Singh, Shashi Rawat, Swarup Kumar Chakrabarti

**Affiliations:** Indian Council of Agricultural Research-Central Potato Research Institute, Shimla, Himachal Pradesh, India; ICAR - National Research Center on Plant Biotechnology, INDIA

## Abstract

Nitrogen is an important nutrient for plant growth and tuber quality of potato. Since potato crop requires high dose of N, improving nitrogen use efficiency (NUE) of plant is an inevitable approach to minimize N fertilization. The aim of this study was to identify and characterize microRNAs (miRNAs) by small RNA sequencing in potato plants grown in aeroponic under two contrasting N (high and low) regimes. A total of 119 conserved miRNAs belonging to 41 miRNAs families, and 1002 putative novel miRNAs were identified. From total, 52 and 54 conserved miRNAs, and 404 and 628 putative novel miRNAs were differentially expressed in roots and shoots, respectively under low N stress. Of total 34,135 predicted targets, the gene ontology (GO) analysis indicated that maximum targets belong to biological process followed by molecular function and cellular component. Eexpression levels of the selected miRNAs and targets were validated by real time-quantitative polymerase chain reaction (RT-qPCR) analysis. Two predicted targets of potential miRNAs (miR397 and miR398) were validated by 5’ RLM-RACE (RNA ligase mediated rapid amplification of cDNA ends). In general, predicted targets are associated with stress-related, kinase, transporters and transcription factors such as universal stress protein, heat shock protein, salt-tolerance protein, calmodulin binding protein, serine-threonine protein kinsae, Cdk10/11- cyclin dependent kinase, amino acid transporter, nitrate transporter, sugar transporter, transcription factor, F-box family protein, and zinc finger protein etc. Our study highlights that miR397 and miR398 play crucial role in potato during low N stress management. Moreover, study provides insights to modulate miRNAs and their predicted targets to develop N-use efficient potato using transgenic/genome-editing tools in future.

## Introduction

Potato is the most important non-grain food crop in the world. In India, potato crop requires high dose of nitrogen (180–240 kg ha^-1^) to produce high tuber yield (30–40 t ha^-1^) [1]. An excessive use of nitrogen causes environmental pollution and degradation of soil health and water quality [2]. Hence, improving nitrogen use efficiency (NUE) of plant is an important approach, which can utilize less nitrogen (N) to produce equivalent tuber yield. This approach is more important in developing countries where resources are limited like India [2]. To achieve this, although agronomic and soil management techniques have been applied to a great extent to improve NUE in crops, this study aimed to identify microRNAs (miRNAs) involved in regulation of gene expression under N stress metabolism in potato [3, 4].

Plant growth and development is controlled by genes and non-coding regulatory molecules like miRNAs [4]. In fact miRNAs are 20–24 nucleotides molecules and they are involved in many biological processes such as cell signaling and development, biotic and abiotic stresses, DNA methylation, auxin response and so on [5]. Mostly miRNAs originate from non-coding region of gene, however a few miRNAs are reported from retrotransposons [6]. Mature miRNAs are processed from primary-miRNAs, which are further processed to precursor-miRNAs [6]. Several miRNAs families have been found in plants and animals and many of them are conserved across organisms [7].

MicroRNAs play a key role in gene regulation after transcription or repression of translation mechanism [7]. Functional validation of miRNAs shows their usefulness in gene regulation, plant growth and development, for example adaptation to abiotic stresses such as drought in chickpea [8], heat in *Saccharina japonica* [9], cold in eggplant [10] and salinity in rice [11, 12]. Several miRNAs have been characterized in plants, such as miR174 and miR167 are involved in N signaling in root development [13]. MiRNAs like miR156, miR159, miR160, miR162, miR166, miR167, miR169, miR171, miR172, miR319, miR393, miR394, miR396, miR397, miR398, miR399 and miR408 have been reported to regulate plant growth under N deficiency [14]. Furthermore, up-regulated miR156/157, miR160, miR164 and miR167; and down-regulated miR169, miR397, miR398, miR399 and miR408 are known under N starvation in plants [15]. Similarly, Shahzad and co-workers [16] reviewed miRNAs function in plant species towards nutrient acquisition and plant adaptation under nutrient stress. In potato, conserved miRNAs families (e.g. miR156, miR397, miR398, miR482, miR5303) have been identified by small RNA sequencing [17]. Besides, characterization of miRNAs and their target genes have also been investigated in Solanaceae plants including potato [18, 19, 20].

With the advancement in next-generation sequencing technologies and availability of the potato genome sequence [21], it is possible to discover miRNAs and target genes in potato. Very recently, we have identified genes under low N stress in potato through RNA-sequencing [22]. Genome-wide identification and characterization of miRNAs in potato is essential to widen our knowledge on gene regulation under low N stress. Hence, in this study we sequenced small RNA to identify conserved and novel miRNAs in response to low N stress versus high N (control) in potato plants grown in aeroponic under controlled conditions. Further, we also performed expression profiles of selected miRNAs and their targets, and 5’RLM-RACE to validate the predicted targets. Our results would enhance knowledge on function of miRNAs in response to adaptation to low N stress in potato.

## Results

### Small RNA sequencing and analysis

**Fig 1:**
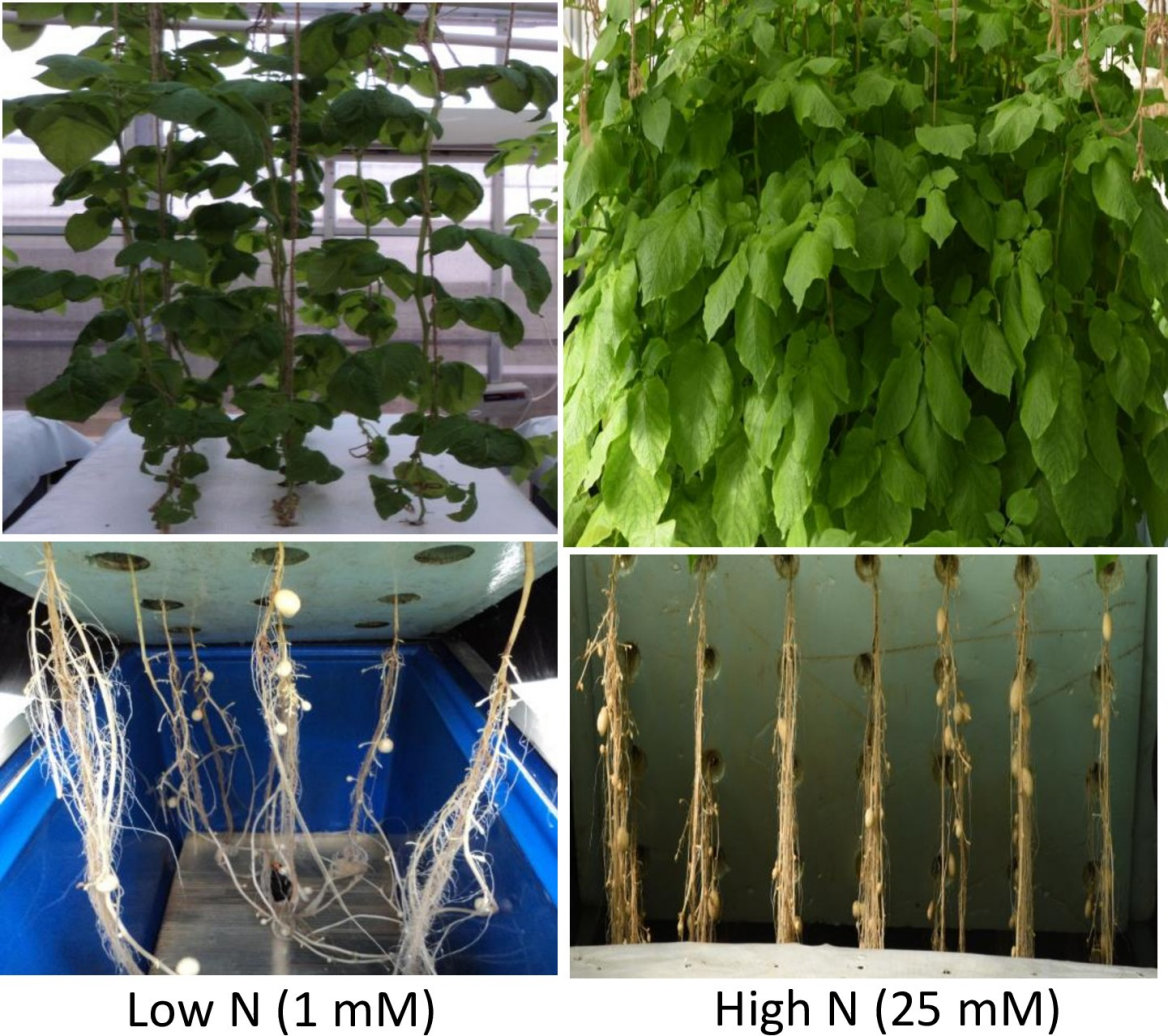
Phenotypes of potato plants grown in aeroponic culture supplied with two contrasting nitrogen regimes (low N: 1 mM, and high N: 25 mM) showing higher plant biomass in high N than low N.

Total RNA was isolated from root and shoot tissues of potato cv. Kufri Jyoti grown in aeroponic under two N (low N and high N-control) regimes (**Fig 1**). Single-end (1 x 75) small RNA libraries were prepared directly from the QC (quality control) passed total RNA. Size of the libraries was ranged between 149–156 bp and sequencing was performed using NextSeq500 (Illumina, San Diego, CA, USA). Nearly 11–21 millions raw reads per sample were generated (**S1**A **Table**), of which nearly 9–18 million reads were recovered after adapter trimming (**S1**B **Table**), from which high quality reads (8–18 million reads per sample) were used for further analysis (**S1**C **Table**). High quality reads with an average read length 23–24 nt and unique tags (2.6–3.9 million per sample) (**S1**D **Table**) were mapped to the Rfam database, and mapped reads, unmapped reads and unique tags were filtered (**S1**E **Table**). Further, unmapped reads and unique tags were mapped to the potato genome sequence repeat database and then filtered (**S1**F **Table**). Reads and unique tags, which were unmapped to the repeat database, were filtered for length distribution (15–34 nt) (**S1**G **Table**). Finally, reads having length more than 34 nt were excluded from analysis and reads size of 15–34 nt were retained for miRNAs identification. Bioinformatics analysis flow is depicted in **S1 Fig**, and mapping of reads to the Rfam database for sample-wise family distribution of miRNAs is shown in **S2 Fig**.

### Identification of conserved and novel miRNAs

**Fig 2:**
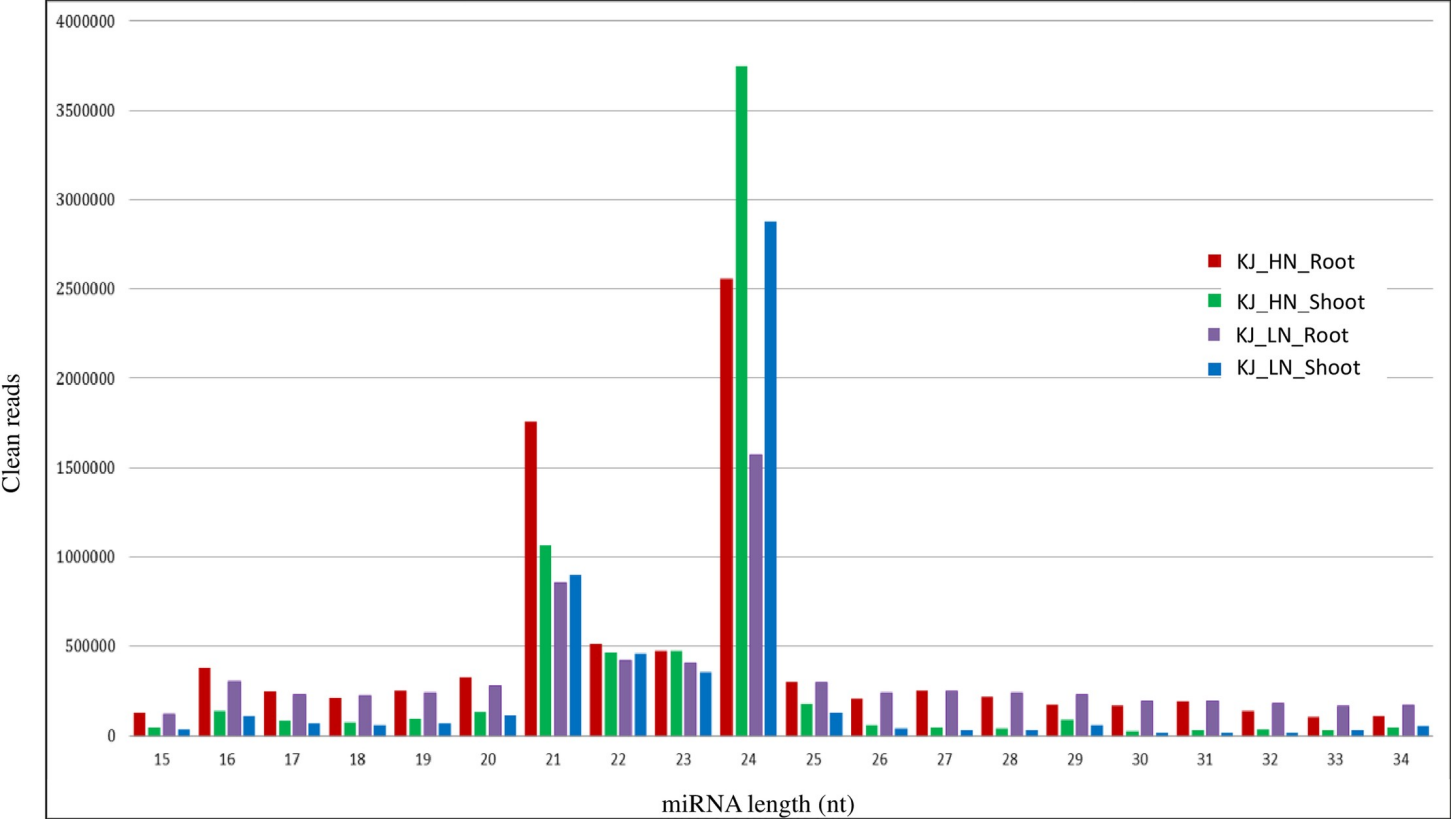
Length distribution of cleaned reads of miRNAs by small RNA sequencing in potato under low N and high N conditions exhibiting maximum miRNA s are of 24 nucleotides (nt) length followed by 21 nt.

Clean reads (15–34 nt) were used for mapping with the potato genome sequence database and alignment summary is outlined in **Table 1** and shown in **Fig 2**. Precursor sequences were identified by the tool miRCat (The UEA small RNA workbench, v 3.2) and only miRNAs observed on stable precursor sequences are reported. After identifying mature sequences, candidate miRNAs were compared with the miRBase ID to assign miR families and then conserved and putative novel miRNAs were segregated (**Table 2**). A total of 119 conserved miNRAs and 1002 putative novel miRNAs were identified in root and shoot tissues in response to low N versus high N (control) (**S2 Table** and **S3 Table**). Number of conserved miRNAs were 110, 105, 108 and 103 in KJ_HN_Root, KJ_LN_Root, KJ_HN_Shoot and KJ_LN_Shoot, respectively (**S4 Table**; KJ: Kufri Jyoti, HN: high N; LN: low N); whereas 615, 381, 890 and 708 putative novel miRNAs were found in KJ_HN_Root, KJ_LN_Root, KJ_HN_Shoot and KJ_LN_Shoot, respectively (**S5 Table**). Maximum 20 conserved miRNAs were identified in potato chromosome 3 followed by chromosome 6 (18), whereas maximum putative novel miNRAs were identified in chromosome 4 (103) followed by chromosome 1 (91) (**Table 3**).

**Table 1:** Alignment summary statistics of small RNA sequencing of potato under low N and high N.

Sample	# Raw reads	# Clean reads*	# Aligned reads
KJ_HN_Root (control)	21,929,322	8,710,979	3,796,038
KJ_LN_Root	21,693,480	6,820,194	2,049,715
KJ_HN_Shoot (control)	14,873,782	6,904,591	4,328,501
KJ_LN_Shoot	11,955,717	5,464,795	3,455,941

*Clean reads (size: 15–34 nt) were obtained from raw reads after removal of adapter trimming, quality trimming, read tag unification, filtering by mapping to Rfam database and repeat database, and read length filtration. KJ: cv. Kufri Jyoti; HN: High nitrogen; LN: Low nitrogen

**Table 2:** Identification of conserved and putative novel miRNAs in potato under low N and high N.

Sample	# Conserved miRNAs	# Putative novel miRNAs
KJ_HN_Root (control) vs.	52	391
KJ_LN_Root	47	233
KJ_HN_Shoot (control) vs.	52	585
KJ_LN_Shoot	47	451

cv. Kufri Jyoti; HN: High nitrogen; LN: Low nitrogen

**Table 3:** Distribution of identified miRNAs on potato chromosomes.

Chromosome	miRNAs	
	Conserved	Putative novel
Chr00	1	-
Chr01	8	91
Chr02	8	73
Chr03	20	72
Chr04	5	103
Chr05	5	66
Chr06	18	87
Chr07	12	53
Chr08	13	69
Chr09	9	90
Chr10	2	68
Chr11	10	63
Chr12	7	72
ChrUn	1	71
Chrx	-	24

Of total 119 conserved miRNAs belonging to 41 miRNAs families, 28 families were found in all four samples, whereas 13 families were tissues-specific (**S6 Table**). We observed miR5303 having largest members (15) followed by miR169 (11 members), miR166 (7 members), miR319 (7 members), and miR172, miR7981 and miR8039 (6 members in each) and other families had 1 to 4 members (**S6 Table**). Whereas, 1002 putative novel miRNAs also had 391, 233, 585 and 451 members in KJ_HN_Root, KJ_LN_Root, KJ_HN_Shoot and KJ_LN_Shoot samples, respectively (**S7 Table**).

**Fig 3:**
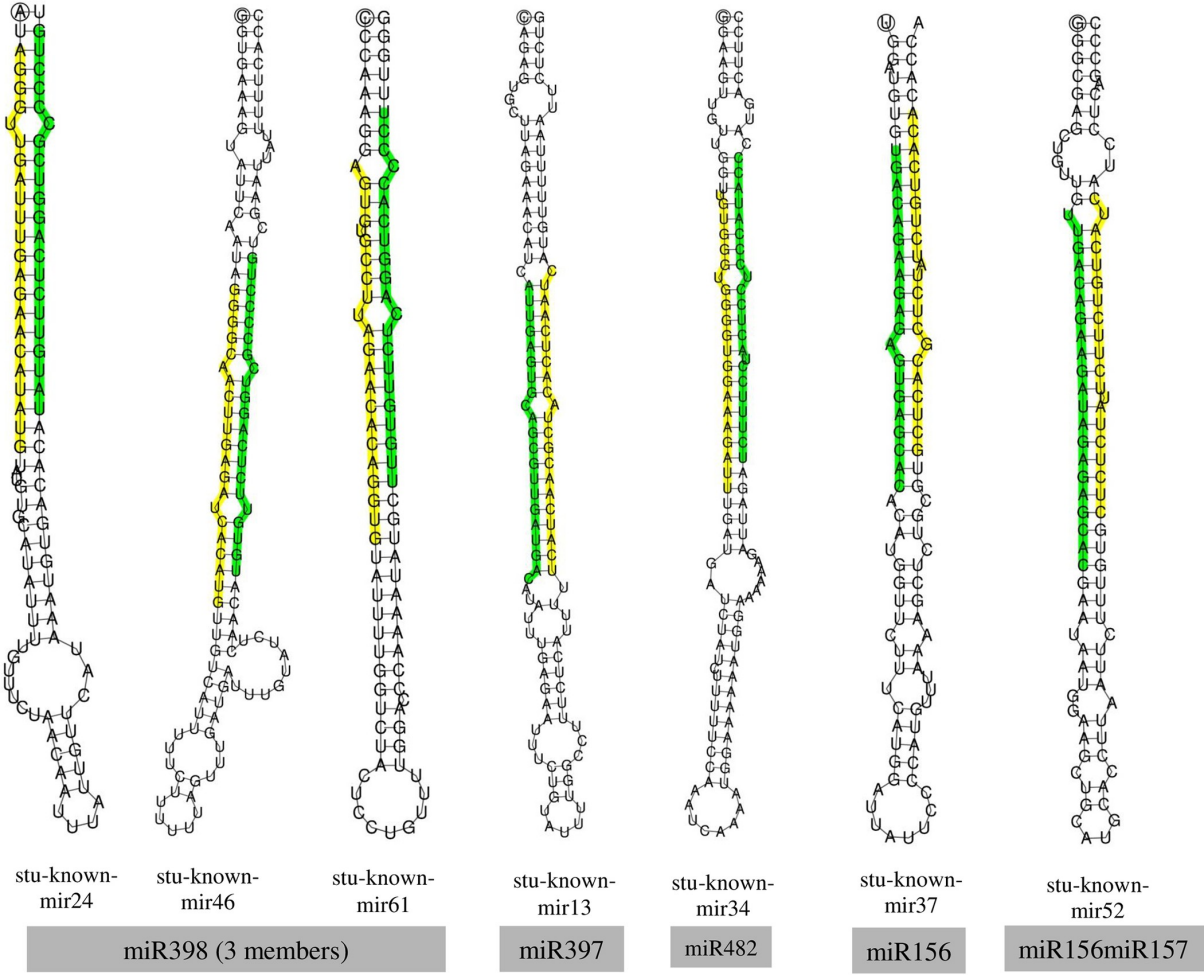
Secondary structures of selected precursor miRNAs identified under low N stress in potato. Sequences which are highlighted with green colour show mature miRNA, whereas yellow colour shows their complementary miRNA* sequences.

**Fig 4:**
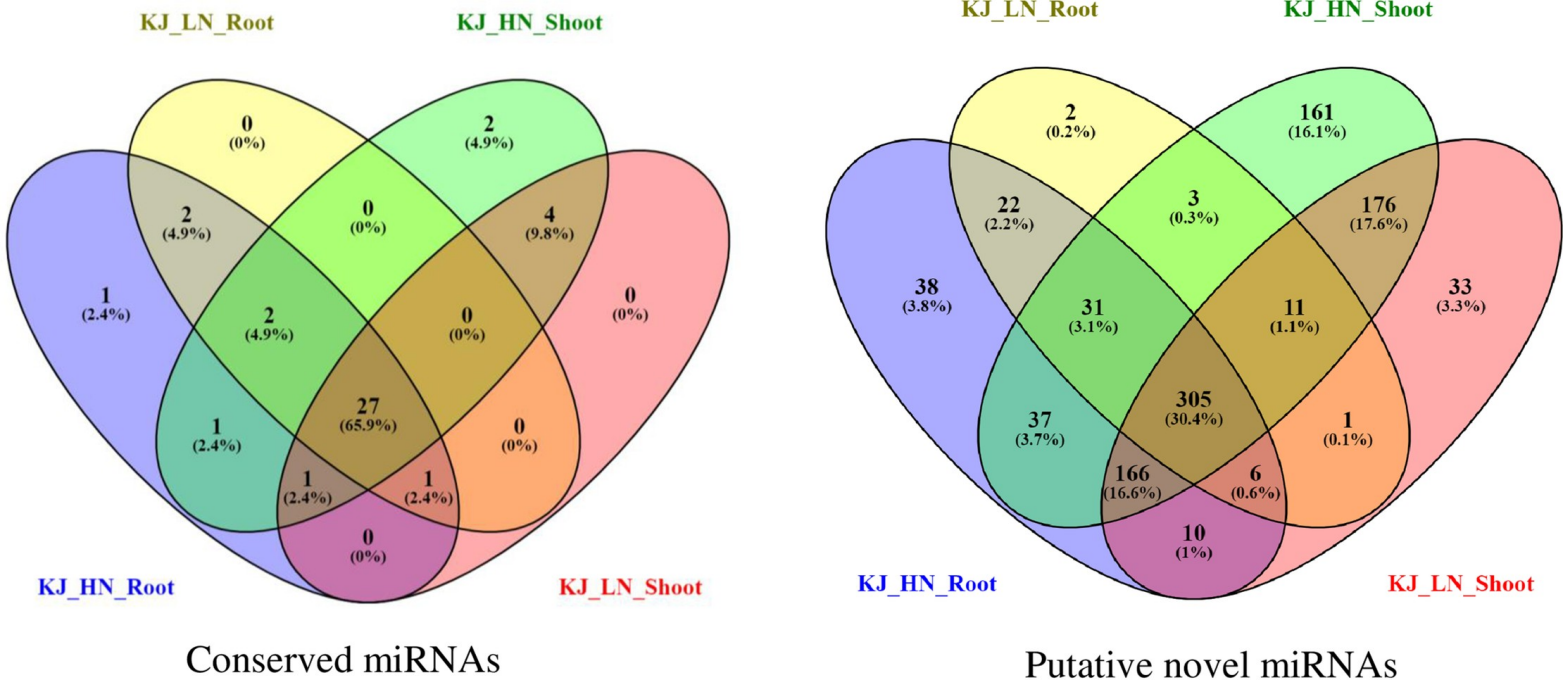
Venn diagram showing distribution of conserved and putative novel miRNAs in root and shoot tissues of potato cv. Kufri Jyoti (KJ) grown in aeroponics with high N (HN) and low N (LN).

Size distribution of conserved miRNAs was ranged between 20–24 nt (miRNAs #19: 20 nt; #63: 21 nt; #11: 22 nt, and #26: 24 nt). Whereas, putative novel miRNAs were ranged between 15–25 nt, of which miRNAs #6: 15 nt, #13: 16 nt, #2: 18 nt, #6: 20 nt, #93: 21 nt, #76: 22 nt, #4: 23 nt, #800: 24 nt, and #2: 25 nt. An average MFE (minimum free energy) of pre-miRNAs of conserved miRNAs was -53.2 ranging between -114.9 to -19.9 kcal mol^-1^, of which only 70 had complementary miRNA* sequences. An average MFE of pre-miRNAs of putative novel miRNAs was -45.9 ranged between -166.4 to -15.4 kcal mol^-1^, of which only 300 had complementary miRNA* sequences. Secondary structures of precursor miRNAs were predicted and highlighted respective mature miRNAs and mature* wherever available. Selected miRNAs are shown in **Fig 3**. Venn diagram analysis showed that 27 (65.9%) conserved miRNAs, and 305 (30.4%) putative novel miRNAs were common among the tissues studied **(Fig 4)**.

### Differential expression analysis of miRNAs

Read counts were used for expression profiling of conserved and putative novel miRNAs. Raw counts were normalized by using TPM (Transcripts Per Million) normalization method and further used to analyze differential expression analysis of miRNAs. Only significantly (*p* < 0.05) expressed miRNAs are reported and summarised in **Table 4**. Of total 119 conserved miRNAs belonging to 41 miRNAs families, 52 miRNAs were differentially expressed in roots, of which 38 were up-regulated, 9 were down-regulated and 5 miRNAs were tissue-specific (KJ_HN_Root) **(S8 Table)**. Whereas, 54 conserved miRNAs were differentially expressed in shoots, 32 were up-regulated, 13 were down-regulated and 9 were tissue-specific (7: KJ_HN_Shoot, and 2: KJ_LN_Shoot) (**S9 Table**). Of total 1002 putative novel miNRAs, 404 were differentially expressed in roots, 92 were up-regulated, 128 were down-regulated and 184 were tissue-specific (171: KJ_HN_root, and 13 KJ_LN_root) (**S10 Table**). Whereas, 628 putative novel miRNAs were differentially expressed in shoots, of which 252 were up-regulated, 156 were down-regulated and 220 were tissue-specific (177: KJ_HN_Shoot, and 43: KJ_LN_Shoot) (**S11 Table**).

**Table 4:** Summary of differentially (*p* < 0.05) expressed miRNAs in potato under low N and high N.

Samples	Known miRNAs		Novel miRNAs	
	Up-regulated	Down-regulated	Up-regulated	Down-regulated
KJ_LN_Root vs. KJ_HN_Root (control)	38	9	92	128
KJ_LN_Shoot vs. KJ_HN_Shoot (control)	32	13	252	156

In addition, several miRNAs were expressed exclusively in tissue specific. cv. Kufri Jyoti; HN: High nitrogen; LN: Low nitrogen

MiR398 and miR397 were most down-regulated in roots by -5.2 and -2.4 log_2_ fold change (FC), respectively; whereas miR5303 and miR398 were down-regulated (-1.2 and -1.1 log_2_ FC, respectively) in shoots under low N compared to high N (control). MiR482 and miR156miR157 were up-regulated (1.7 and 1.6 log_2_ FC, respectively) in roots, whereas miR319 and miR156 were up-regulated (both 1.8 log_2_ FC) in shoots. In terms of most up-regulation, putative novel miRNAs stu-novel-mir963 (2.9 log_2_ FC) and stu-novel-mir1052 (2.6 log_2_ FC) showed higher expression in roots and shoots, respectively.

### Target prediction of miRNAs and GO characterization

**Fig 5:**
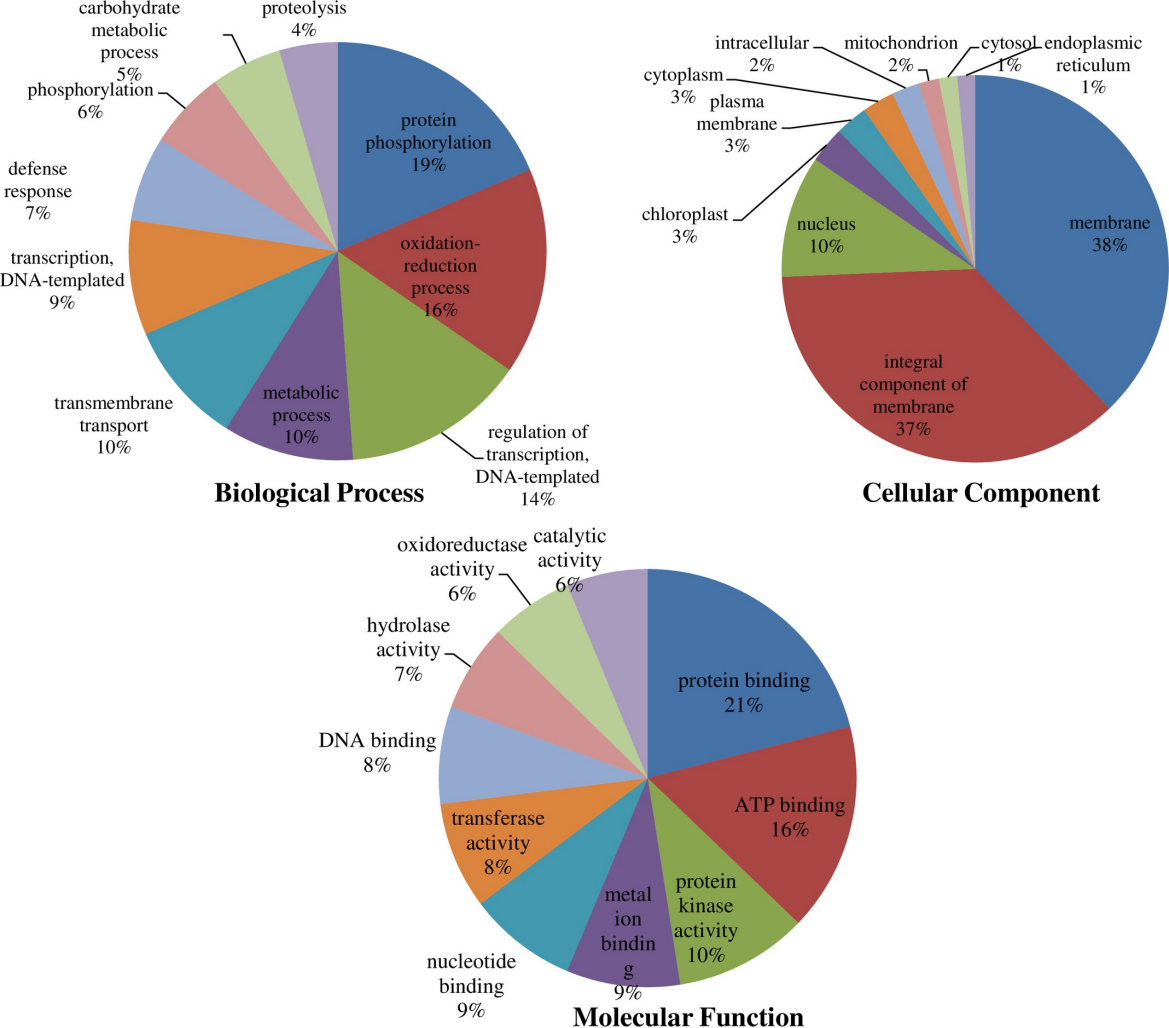
GO characterization of predicted target genes of miRNAs of top ten GO terms in each biological process, cellular component and molecular function categories.

The psRNATarget finder tool was used to predict targets of miRNAs. A total of 34,135 targets were predicted by 723 miRNAs (61 conserved and 662 putative novel miRNAs) (**S12 Table**). In-silico targets finding showed that most miRNAs regulate gene expression by cleavage mechanism followed by inhibition of translation process. Predicted targets were characterized by total 4399 GO terms, of which 2327 were categorised to biological process, 1583 were belonged to molecular function, 488 were under cellular component, and one was of ‘eco’ category (**S13 Table**). Based on the GO analysis, we predicted several target genes such as Cdk10/11- cyclin dependent kinase (49 miRNAs), amino acid transporter (29 miRNAs), receptor protein kinase (28 miRNAs), F-box domain containing protein (27 miRNAs), cytochrome P450 (27 miRNAs), serine/threonine-protein kinase PBS1 (23 miRNAs), F-box family protein (21 miRNAs), ankyrin repeat-containing protein (21 miNAs) and many unknown genes. In addition, many unknown transcripts were targeted by miRNAs like PGSC0003DMT400044989 (50 miRNAs) and PGSC0003DMT400004014, PGSC0003DMT400044986 and PGSC0003DMT400044988 (each 49 miRNAs). Based on hits value, top five GO numbers were belonged to cellular component (GO:0016020, hits: 16638, membrane; and GO:0016021, hits: 16073, integral component of membrane) followed by molecular function (GO:0005515, hits: 9268, protein binding; and GO:0005524, hits: 7118, ATP binding) and biological process (GO:0006468, hits: 4585, protein phosphorylation). Selected top ten GO terms of the predicted targets of miRNAs is depicted in **Fig 5**.

### Validation of miRNAs expression by RT-qPCR analysis

**Fig 6:**
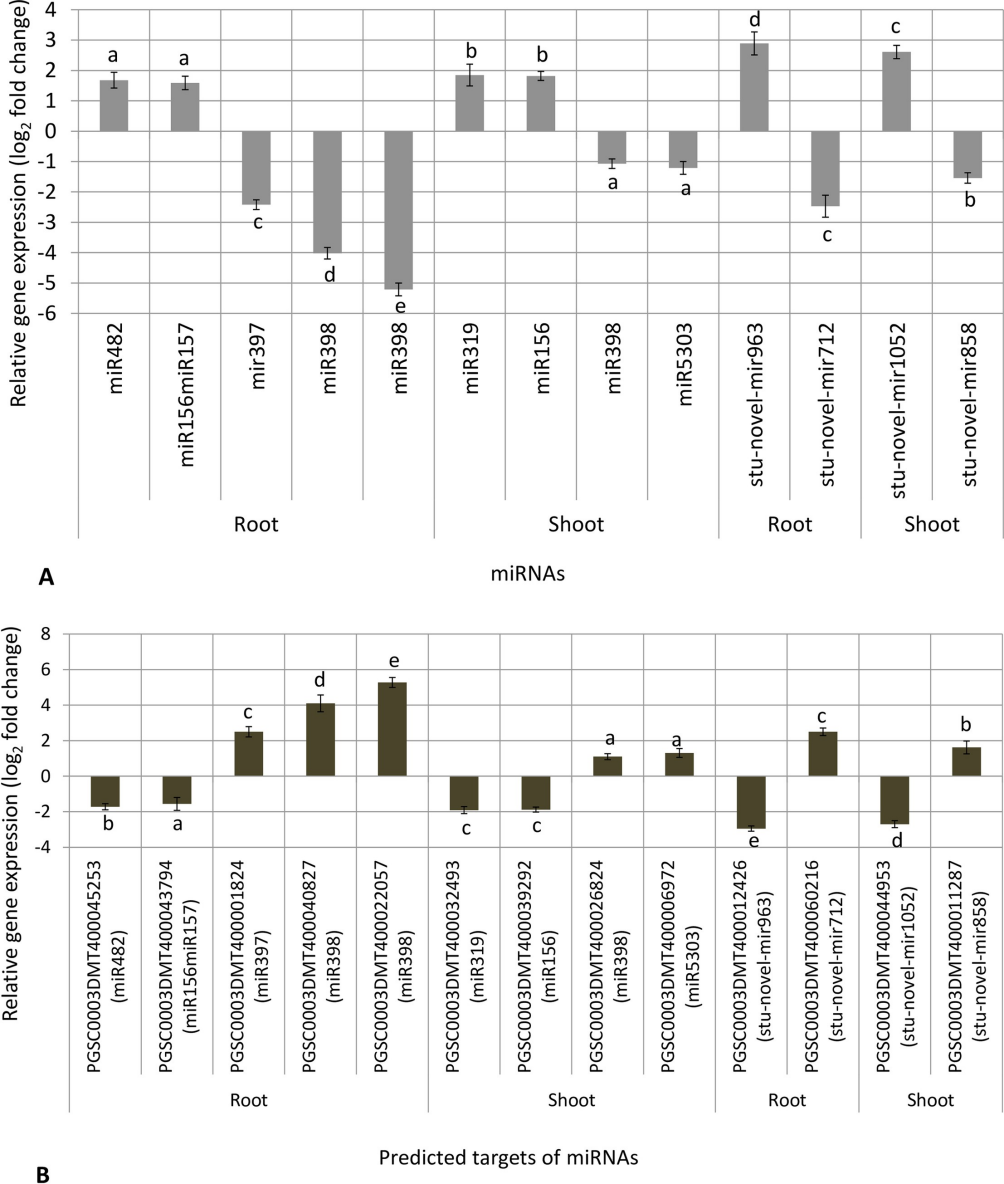
Validation of miRNAs (A) and their predicted targets (B) by relative gene expression (log_2_ fold change) by RT-qPCR analysis. Small letter on bar with different alphabet indicates significance at *p* < 0.05, whereas same alphabet shows non-significance within up-regulated or down-regulated miRNAs/targets. Values are means ± standard error.

Selected miRNAs were validated by RT-qPCR expression analysis and results showed good concordance with small RNA sequencing profile **(Fig 6(A))**. Among the conserved miRNAs in roots under low N stress, stu-known-mir34 (miR482) and stu-known-mir52 (miR156miR157) were up-regulated (1.68 and 1.59 log_2_ FC, respectively), whereas stu-known-mir46 (miR398), stu-known-mir24 (miR398) and stu-known-mir1 (miR397) were most down-regulated (-5.21, -4.02 and -2.42 log_2_ FC, respectively). Besides, among the conserved miRNAs in shoots under low N, stu-known-mir57 (miR319) and stu-known-mir37 (miR156) were both up-regulated 1.85 log_2_ FC; and stu-known-mir61 (miR398) and stu-known-mir14 (miR5303) were most down-regulated (-1.07 to -1.21 log_2_ FC, respectively). Whereas among putative novel mRNAs under low N stress in roots, stu-novel-mir963 was most up-regulated (2.89 log_2_ FC) and stu-novel-mir712 was most down-regulated (-2.47 log_2_ FC); and stu-novel-mir1052 was most up-regulated (2.61 log_2_ FC) and stu-novel-mir858 was most down-regulated (-1.54 log_2_ FC) in shoots **(S14 Table)**.

### Expression analysis of targets by RT-qPCR

Gene expression of predicted targets was analyzed by RT-qPCR analysis, which showed inverse expression profiles as compared to miRNAs **(Fig 6(B))**. A few targets of miRNAs were namely universal stress protein family protein for stu-known-mir34 (miR482); nitrate transporter for stu-known-mir52 (mir156mir157); calmodulin-binding heat-shock protein for stu-known-mir24 (miR398); heat shock protein binding protein for stu-known-mir46 (miR398), nitrate transporter for stu-known-mir57 (miR319); amino acid transporter for stu-known-mir37 (miR156); serine-threonine protein kinase, plant-type for stu-known-mir61 (miR398); F-box family protein for stu-known-mir14 (miR5303); transcription factor for stu-novel-mir963; zinc finger protein for stu-novel-mir712; amino acid transporter for stu-novel mir1052; and sugar transporter for stu-novel-mir858 **(S14 Table)**.

### Target validation of conserved miRNAs by 5’ RLM-RACE

**Fig 7:**
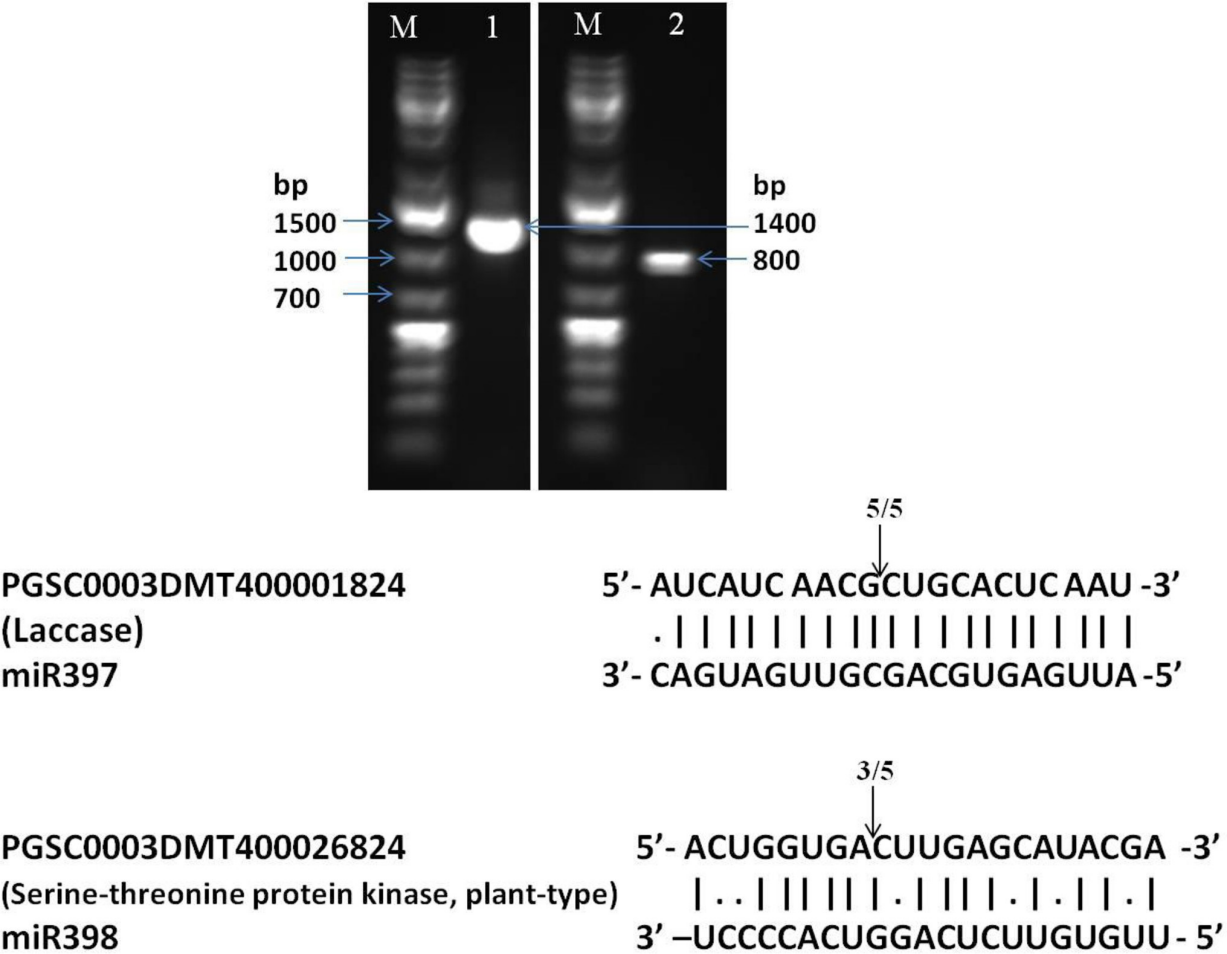
Target validation of genes 1) PGSC0003DMT400001824 (Laccase) of the miR397 in root, and 2) PGSC0003DMT400026824 (Serine-threonine protein kinase, plant-type) of the miR398 in shoot by 5’RLM-RACE. Agarose gel (1%) image of 5’-RLM-RACE products, and targets and miRAs sequences are shown here. Cleavage site of two conserved miRNAs is shown by the arrow with the frequency of cloned RACE products above it. The vertical lines indicate match base pairs and dot (.) indicate mismatch. M = 1 kb DNA ladder.

Two targets (one each) of conserved miRNAs (miR397 and miR398) were validated by 5’RLM-RACE. The predicted targets PGSC0003DMT400001824 (Laccase) of miR397 and PGSC0003DMT400026824 (Serine-threonine protein kinase, plant-type) of miR398 were amplified with distinct band (**Fig 7)**. Further, the amplified 5’ RLM-RACE products were eluted, cloned and sequenced to analyse cleavage pattern based on sequence complementarity between miRNAs and mRNA (target) (**Fig 7).**

## Discussion

Improving NUE of potato plant is essential to reduce amount of N fertilizers applied to the crop, while maintaining tuber yield. This approach could reduce production cost and save environment from excessive use of N [2]. We aimed to discover miRNAs regulating gene networks in potato under N stress. Very recently we have shown precision phenotyping of potato under varied N supplies in aeroponic under controlled conditions [2] and identified genes under N stress [22], however genome-wide identification of miRNAs is yet to be investigated. In this study, we identified 119 conserved miRNAs (20–24 nt) belonging to 41 miRNAs families, and 1002 putative novel miRNAs (15–25 nt) responsive to low N stress in potato. The differential expression analysis of conserved miRNAs shows that miR397 and miR398 play crucial role in potato under low N and their targets were also validated in the study. The GO analysis indicates that maximum target genes belong to biological process followed by molecular function and cellular component. However, the highest number of hits of miRNAs to targets were observed with cellular component (membrane, and integral component of membrane), indicating their role during adaptation to N stress in potato.

Our findings on miRNAs identification in potato corroborate with earlier research. A few selected targets of miR398 were heat shock protein binding protein, calmodulin-binding heat shock protein, zinc finger protein, cytochrome P450, and serine-threonine protein kinase plant-type. Earlier study shows that miR398 is directly associated with stress regulatory network genes and responses to various stresses like high salt, water deficit, oxidative, abscisic acid, phosphorpus and copper deficiency, high sucrose and bacterial infection [23]. Trindade et al. [24] revealed up-regulation of miR398 and miR408 in response to water deficit in *Medicago truncatula*. Manipulation of microRNA expression has been investigated in plants, such as miR397 and miR398 were down-regulated, and miR156/157 were up-regulated under N starvation [15]. Similarly, Shahzad et al. [16] reviewed role of miRNAs in nutrient acquisition and plant adaptation under N stress and reported down-regulated miR169, miR397 and miR398, and up-regulated miR156, miR157 and miR169. In this study, miR397 modulated target genes such as universal stress protein, zinc finger protein, heat shock protein and serine-threonine protein kinase, laccase and root border cell-specific protein. Study indicates that miR397 is conserved in both dicot and monocot plant species and laccase is a target gene [25]. Most research work focussed on abiotic stress response of miR397 in plants, such as up-regulation under drought stress in rice [26], and up-regulation under cold, drought, and high salinity stresses in *Arabidopsis* [27]. Role of miR397/laccase gene has also been demonstrated in improving tolerance to fenoxaprop-P-ethyl in *Beckmannia syzigachne* and *Oryza sativa* [28]. Zuluaga et al. [29] identified miR397 were found under N starvation in wheat at grain filling stage. Thus, these studies illustrate importance of miR397 and miR398 in plant metabolism, and our study observed their role in potato adaptation to low N stress.

In this study, miR156/157 encoded several targets such as nitrate transporter and salt-tolerance protein; whereas amino acid transporter and serine-threonine protein kinase plant-type are some predicted targets of miR156. MiR156 is one of the first microRNAs described in *Arabidopsis* by small RNA sequencing [30]. MiR156 is a graft-transmissible miRNA that modulates plant architecture and tuberization in *Solanum tuberosum* ssp. *andigena* [31]. Zeng et al. [14] reviewed role of various microRNAs in plant development in response to N deficiency. A few miRNAs identified in our study corroborate with earlier finding such as miR156 and miR319 (shoot development), miR393 (root development, defense response), miR397 (copper homoeostasis, lignin synthesis), and miR398 (copper homeostasis, oxidative stress) [14]. Song et al. [32] identified miRNAs such as miR156 in *Chrysanthemum nankingense* under N starvation condition. Moreover, role of miR156 has also been demonstrated in modulation of traits associated with vegetative phase in tobacco [33] and other plants [34]. MiR156-regulated traits therefore can be used to distinguish between juvenile and adult phases of development in tobacco and in the family Solanaceae. Zhang et al. [17] identified 28 conserved miRNAs families such as miR156, miR319, miR397, miR398, miR482, and miR5303 in potato by small RNA sequencing. MiR482 encoded target genes such as universal stress protein family protein, transcription factor and sugar transporter in potato under N stress. MiR482/2118 family has shown to regulate nucleotide binding site (NBS) and leucine-rich repeat (LRR) class of resistance genes. Zhu et al. [35] demonstrated expression of NBS-LRR genes by miR482 mediated gene silencing pathway upon attack of fungus (*Verticillium dahlia*). Shivaprasad et al. [36] illustrated miR482 mediated regulation of NBS-LRR motifs proteins for disease resistance in tomato. Similarly, de Vries et al. [37] investigated regulation of *Phytophthora resistance* by miR482/2118 expression in wild and cultivated tomatoes. Numerous such findings evidence role of miRNAs in plant stress management, and we observed their implication to manage N stress in potato.

Besides, in this study, miR319 targeted genes like nitrate transporter, amino acid transporter and transcription factor. Moreover, miR319 regulates robust and multilayer control of leaf development in *Arabidopsis* and crop plants [38]. Kondhare et al. [39] evidenced role of miR319 and miR482 etc. in early stage of stolon initiation and tuberization process in potato under short-days and long-days conditions. MiR319 also plays an important role in gibberellins network to confer immune response to *Potato Virus Y* infection in potato [40]. Whereas, F-box family protein, ATP binding protein, and syntaxin are some target genes of miR5303. Gu et al. [41] outlined several miRNAs including miR5303 in six Solanaceous plants (potato, tomato, tobacco, eggplant, pepper, and petunia) and showed their potential association with phosphate and mycorrhizal signalings. Khaldun et al. [42] investigated miRNAs like miR156, miR397, miR398, miR5303 etc. and their target genes in the fruit and shoot tip of *Lycium chinense*, a traditional Chinese medicinal plant. Hou et al. [43] suggest the involvement of miR156, miR319, miR482, miR5303 etc. families in development of long non-coding RNAs during tuber sprouting process of potato. In addition to the above conserved miRNAs families, miR5303 contained 15 members followed by miR169 contained 11 members; miR166 and miR319 contained 7 members; and miR172, miR7981 and miR8039 contained 6 members, and rest 34 conserved miRNAs contained 1–3 members, depending upon the tissues used in the study. These miRNAs were also differentially expressed in potato under low N stress. MiR169 targets genes like *Aspartate aminotransferase* and transcription factors (YA1, YA6, YA3, YA4, and BZIP transcription factor BZI-2). MiR169 isoforms regulate root architecture in *Arabidopsis* and their targets are linked to nutrient signalling in plants [44]. Role of graft-transmissible miR172 in induction of potato tuberization has been proven by Martin et al. [45]. Thus, researchers have evidenced role of miRNAs in plant metabolism and manifested their relevance under low N stress in potato.

**Fig 8:**
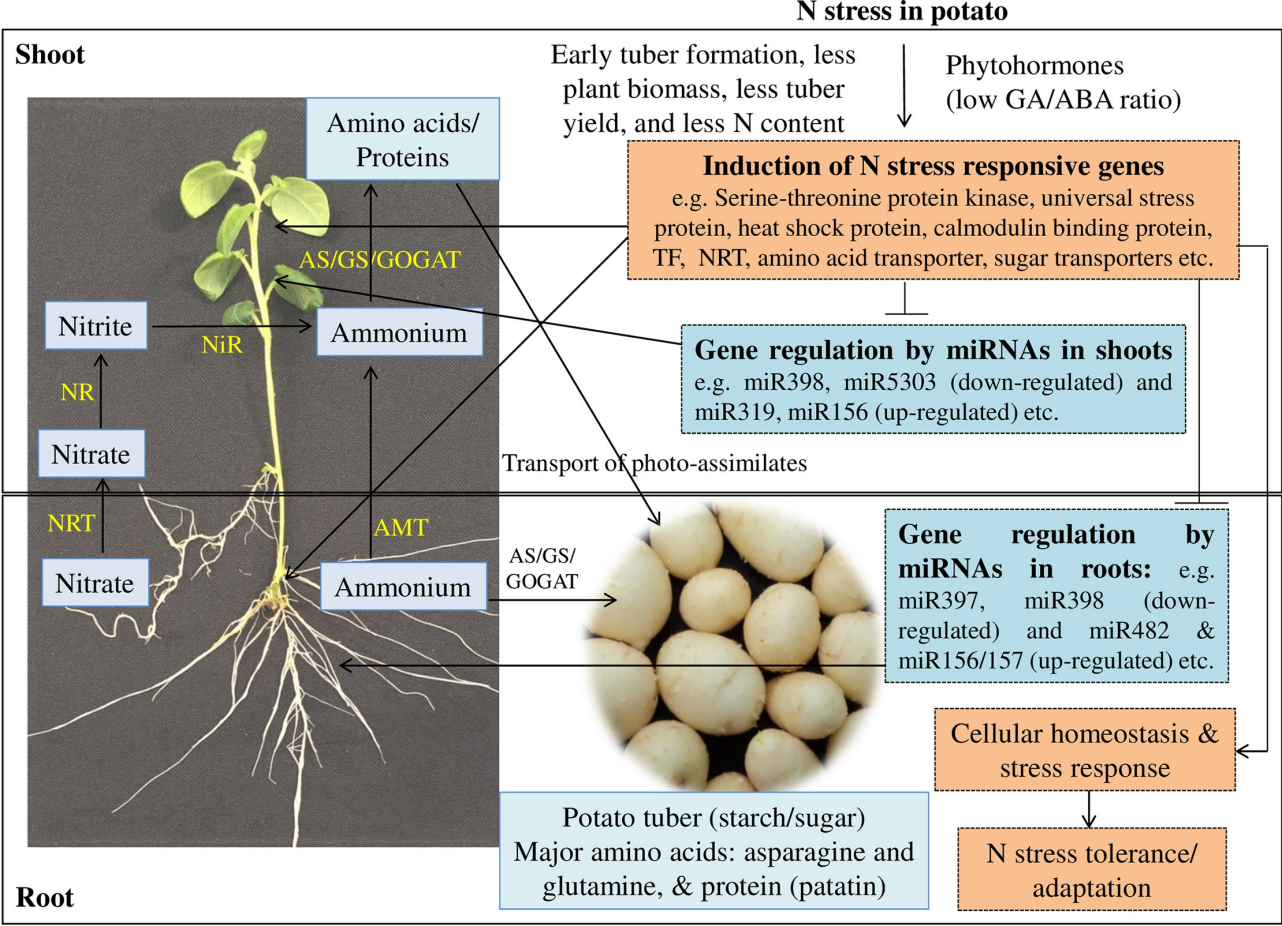
Schematic presentation of identification of miRNAs and their targets under N stress metabolism in potato. *‘T’* head sign shows repression of targets by miRNAs and arrow indicates induction or association. NR: Nitrate reductase, NiR: Nitrite reductase, AS: Asparagine synthaease, GS: Glutamine synthetase; GOGAT: glutamine oxoglutarate amino transferase; NRT: Nitrate transporter; AMT: Ammonium transporter.

Taken together, our study substantiates that several miRNAs play a key role in adaptation to low N stress in potato. Of which, we have confirmed that miR397 and miR398 play a critical role during N stress metabolism in potato. A schematic diagram of selected miRNAs and their potential target genes involved in N metabolism in potato are depicted (**Fig 8**). Further investigation is required to modulate expression of miRNAs and their targets to develop N-use efficient potatoes which would adapt to low N stress without compromising yield applying modern genomics tools like genome editing.

## Materials and methods

### Plant materials and N treatments

An Indian potato variety Kufri Jyoti (KJ) was used in this study, which is N inefficient [46]. Healthy in vitro plants were routinely maintained at Indian Council of Agricultural Research-Central Potato Research Institute, Shimla (31.1048° N, 77.1734° E 2,276 m above mean sea level), Himachal Pradesh, India. In vitro plants were grown in aeroponic till full crop cycle (110 days) with two nitrogen treatments [High N (HN): 25 milli molar N; and Low N (LN): 1 milli molar N] using both nitrate and ammonium form of N under controlled conditions supplied with all major and minor nutrients solution (except N) as described by Tiwari and co-workers [46] (**Fig 1**). Ammonium form of N was limited to 0.5 mM in both the treatments. Shoot and root samples (three replicates) were collected from 45 days old plants of both high and low N treatments and processed for small RNA sequencing for samples namely ‘KJ_LN_Shoot’ versus ‘KJ_HN_Shoot’ (control); and ‘KJ_LN_Root’ versus ‘KJ_HN_Root’ (control).

### RNA isolation, library preparation and sequencing

Total RNA was isolated from root and shoot samples by modified c-TAB and lithium chloride method [47]. Quality and quantity of isolated RNA samples were checked on 1% denaturing RNA agarose gel and NanoDrop, respectively. Small RNA sequencing libraries (single end, read length: 1 x 75 bp) were prepared directly from the isolated QC passed total RNA using TruSeq Small RNA Library Preparation Kit as per the manufacturer’s instruction (Illumina, San Diego, CA, USA). The gel size selected and purified libraries were analyzed on 4200 Tape Station system (Agilent Technologies, Santa Clara, CA, USA) using High sensitivity D1000 Screen Tape and then libraries were used for sequencing in NextSeq 500 following manufacturer’s instruction (Illumina, San Diego, CA, USA). Details of analysis with parameters setting are described in **S1 File**.

### Data processing and analysis

Raw reads were screened for adapter contamination using the Cutadapt tool (v 1.16) to retain only putative miRNAs (Phred quality score: QV 20) [48]. Then quality filtering analysis was performed to remove any low quality read or trim terminal low quality bases using the Trimmomatic tool (v 0.38) [49]. Adapter trimmed and quality filtered clean reads were formatted into a non-redundant fasta format. Occurrence of each unique sequence read was counted as sequence tag. Number of reads for each unique tag reflects relative expression level of miRNA. Considering tags instead of reads allows a high loss-less compression of original data and simplifies computational analysis.

Since, small RNA sequencing data is known to include reads from other category of small non-coding RNAs, read tags were blasted against customized Rfam database of Rfam (v13) [50]. Read tags, which were not mapped to the Rfam, were mapped to known repeat sequences i.e. Repbase (v 22) using NCBI Blast and any tag matched with above mentioned stringent criteria was excluded from further analysis. Finally, reads having length more than 34 nt (nucleotide) were also excluded from analysis. Adapter trimmed, quality filtered and length filtered reads were considered as cleaned putative miRNAs population and were used for miRNAs identification.

### Reads mapping to the reference potato genome

Clean reads were mapped to the reference potato genome. Precursor sequences were identified by the tool miRCat, The UEA small RNA workbench (v 3.2) [51, 52]. The miRcat analyses reads mapping pattern along with few upstream and downstream bases. It uses RNAfold to estimate the precursor stability by analyzing MFE value. Only miRNAs observed on stable precursor sequences were reported.

### Identification of conserved and novel miRNAs

On identifying mature miRNAs, candidate mature sequences were compared with miRBase (a conserved miRNA database) and assigned with miR families [53]. Thus, conserved miRNAs were identified based on the assigned miRBase ID, and rest others without miRbase ID were categorized as putative novel miRNAs. Unique mature miRNAs sequences found in each sample were tabulated. In-house custom wrapper scripts based on RNAfold was used to generate secondary structure of precursor sequences of miRNAs. Venn diagram was prepared for the identified conserved and putative novel miNRAs using Venny tool [54] with the default setting to identify common and tissue specific miRNAs.

### Differential expression analysis of miRNAs

After aligning with the potato genome, reads count were used for expression profiling of conserved and putative novel miRNAs. miRNAs with at least of 10 reads support in any of the four samples were considered for differential analysis. TPM normalization was applied to normalize reads count of miRNAs population per sample. TPM values were then log (base _2_) transformed to get fold expression values and finally log_2_ FC values were calculated by subtracting respective fold expression values. FC value of above zero is considered as up-regulated, whereas below zero is considered as down-regulated.

### Prediction of miRNAs targets and GO characterization

The psRNATarget finder tool was used to predict potential targets of miRNAs based on unpaired energy (UPE) using default parameters as latest version of the tool (v 2017). Predicted targets were then characterized by the GO terms.

### Validation of miRNA expression by RT-qPCR

RT-qPCR analysis was performed to confirm the expression level of selected miRNAs. Total RNA of same stage (45 days) plants was used to generate cDNA by reverse transcription using Mir-X^™^ miRNA First-Strand Synthesis and TB Green^TM^ RT-qPCR kit following manufacturer’s instructions (Takara Bio USA Inc., CA, USA) in ABI PRISM HT7900 (Applied Biosystems, Warrington, UK). The entire sequence of mature miRNAs was used as miRNA-specific 5’ primer, whereas 3’ primer (mRQ 3’ Primer) was supplied with the kit. Thermal cycles profile of RT-qPCR was followed as: denaturation at 95°C for 10 s; 40 cycles of 95°C for 5 s and 60°C for 20 s; and dissociation at 95°C for 60 s, 55°C for 30 s and 95°C for 30 s. A normalization standard gene U6 snRNA was used to measure relative expression of miRNA by the delta-delta Ct method (^ΔΔ^Ct) [55]. It measures relative expression of miRNAs by comparing with normalization standard U6 snRNA. miRNAs and U6 snRNA were amplified in each sample to determine the Ct for each, by which relative expression was determined using the ^ΔΔ^Ct calculation. RT-qPCR data of miRNAs were analysed in three replications (both biological and technical) and calculated FC using ^ΔΔ^Ct method [55]. Standard errors were calculated from the replicated data and expression result is shown in bar diagram (mean ± standard error). Replicated expression data was analysed with one way analysis of variance (ANOVA) using XLSTAT2018.5 (www.xlstat.com) and means were compared using Tukey’s test at least significance level (*p* < 0.05) [46]. Means of up-regulated and down-regulated miRNAs were analysed separately.

### Expression analysis of targets by RT-qPCR

Total RNA of all four samples of same stage (45 days) was reverse transcribed using TaqMan® Reverse Transcription Reagent kit (Applied Biosystems, New Jersey, USA). RT-qPCR primers were designed using Primer Express 2.0 software (Applied Biosystems, CA, USA), as given in **S14 Table**. RT-qPCR was performed using Power SYBR Green PCR Master Mix (Applied Biosystems, Warrington, UK) in the ABI PRISM HT7900 following thermal cycler profiles 50°C for 2 min; 95°C for 10 min; and 40 cycles of 95°C for 15 s, 60°C for 1 min, 72°C for 30 s using internal standard of mitochondrial cytochrome oxidase gene (*cox*I) (X83206.1) as described earlier in potato[56, 57]. RT-qPCR expression data of target genes was analysed as above like miRNAs [46].

### Validation of targets of conserved miRNAs by 5’ RLM-RACE

Two predicted targets (one each) of miRNAs (miR397 and miR398) were validated by 5’ RLM-RACE technique using the SMARTer RACE 5’/3’ Kit following manufacturer’s instruction (Cat. No. 634858; Takara Bio USA, Inc., CA, USA). In brief, poly A^+^ RNA was isolated from same stage (45 days) plant tissues (roots and leaves) using NucleoSpin RNA Plant kit (Cat. No. 740949.50; Takara Bio USA, Inc., CA, USA). Poly A^+^ RNA (1 μg) was processed for first-strand cDNA synthesis, and then 5’ RACE-ready cDNA was used for 5’ RACE amplification using 5’ gene-specific primer and Universal Primer A Mix, as provided with the kit. The PCR cycles used for the RACE amplification were 30 cycles of 94°C for 30 s, 67°C for 30 s and 72°C for 5 min. The PCR products were eluted, cloned in pRACE vector (a pUC19-based vector) and sequenced using M13 universal primer sequences to identify cleavage site. The gene-specific primers were designed using IDT (Integrated DNA technologies, USA) tool and only 5’ primers were used for 5’ RLM-RACE amplification **(S15 Table)**.

## Supporting information

S1 Fig Bioinformatics data analysis workflow of small RNA sequencing. Figure shows bioinformatics work flow of small RNA sequencing and data analysis.
(DOCX)

S2 Fig Abundance of reads mapped to the Rfam database for miRNAs families distribution. miRNAs families distribution for samples: a. KJ_HN_Root; b. KJ_LN_Root; c. KJ_HN_Shoot; d. KJ_LN_Shoot.
(DOCX)

S1 File Supplementary method information of small RNA sequencing and data analysis. Illumina nextseq small RNA library preparation, adapter trimming analysis, quality filtering analysis, uniquification of read tags, reads filtering (removal of known non-coding RNA mapping reads), reads filtering (removal of repeat database mapping reads), reads length filtration, reads mapping to the reference genome, known and novel miRNAs indemnification analysis, differential analysis, precursor sequences secondary structures, miRNAs targets identification analysis.
(DOCX)

S1 Table Details of data generation by small RNA sequencing for N stress in potato. a) raw reads data statistics, b) adapter trimming analysis summary statistics, c) quality trimming analysis summary statistics, d) read tags uniquification summary statistics, e) Rfam database mapped read filtering statistics, f) repeat database mapped read filtering statistics, and g) read length filtration summary analysis.
(DOCX)

S2 Table Total conserved miRNAs in potato under N stress. A list of total 119 conserved miRNAs identified in potato under N stress are summarized in sheets with detailed features.
(XLSX)

S3 Table Total putative novel miRNAs in potato under N stress. A list of total 1002 putative novel miRNAs identified in potato under N stress are summarized in sheets with detailed features.
(XLSX)

S4 Table Sample-wise details of conserved miRNAs in potato under N stress. A list of miRNAs for samples KJ_HN_Root, KJ_LN_Root, KJ_HN_Shoot, and KJ_LN_Shoot.
(XLSX)

S5 Table Sample-wise details of putative novel miRNAs in potato under N stress. A list of miRNAs are summarized in sheets for samples namely KJ_HN_Root, KJ_LN_Root, KJ_HN_Shoot, and KJ_LN_Shoot.
(XLSX)

S6 Table Family members of conserved miRNAs in potato under N stress. A list of miRNAs family members are summarized in sheets for samples namely KJ_HN_Root, KJ_LN_Root, KJ_HN_Shoot, and KJ_LN_Shoot.
(XLSX)

S7 Table Family members of putative novel miRNAs in potato under N stress. A list of miRNAs family members are summarized in sheets for samples namely KJ_HN_Root, KJ_LN_Root, KJ_HN_Shoot, and KJ_LN_Shoot.
(XLSX)

S8 Table Differentially expressed conserved miRNAs in root in potato under N stress. A list of total 52 differentially expressed conserved miRNAs are summarized in sheets in root samples (KJ_LN_Root versus KJ_HN_Root (control).
(XLSX)

S9 Table Differentially expressed conserved miRNAs in shoot in potato under N stress. A list of total 54 differentially expressed conserved miRNAs are summarized in sheets in shoot samples (KJ_LN_Shoot versus KJ_HN_Shoot (control).
(XLSX)

S10 Table Differentially expressed putative novel miRNAs in root in potato under N stress. A list of total 404 differentially expressed putative novel miRNAs are summarized in sheets in root samples (KJ_LN_Root versus KJ_HN_Root (control).
(XLSX)

S11 Table Differentially expressed putative novel miRNAs in shoot in potato under N stress. A list of total 628 differentially expressed putative novel miRNAs are summarized in sheets in shoot samples (KJ_LN_Shoot versus KJ_HN_Shoot (control).
(XLSX)

S12 Table Prediction of target genes of both conserved and putative novel miRNAs identified in potato under N stress condition. A list total 79303 potential target genes of both conserved and putative novel miRNAs in potato are summarized in sheets.
(XLSX)

S13 Table Gene Ontology (GO) characterization of target genes in potato. Summary of GO characterization of target genes of miRNAs identified in potato under N stress are summarized in sheets.
(XLSX)

S14 Table RT-qPCR analysis. Summary of RT-qPCR primers used for gene expression analysis of miRNAs and their corresponding targets.
(DOCX)

S15 Table 5’ RLM-RACE primers and sequences. Primer sequences of 5’ RLM-RACE used for target validation and sequences of cloned product of RACE product.
(DOCX)
