# Supplementary figures and images for: Genome-wide identification and characterization of microRNAs by small RNA sequencing for low nitrogen stress in potato
Source: PLoS One. 2020 May 19;15(5):e0233076. doi: 10.1371/journal.pone.0233076 (PMC7237020; doi:10.1371/journal.pone.0233076)

**Figure S1.**

**Bioinformatics Data Analysis Workflow**


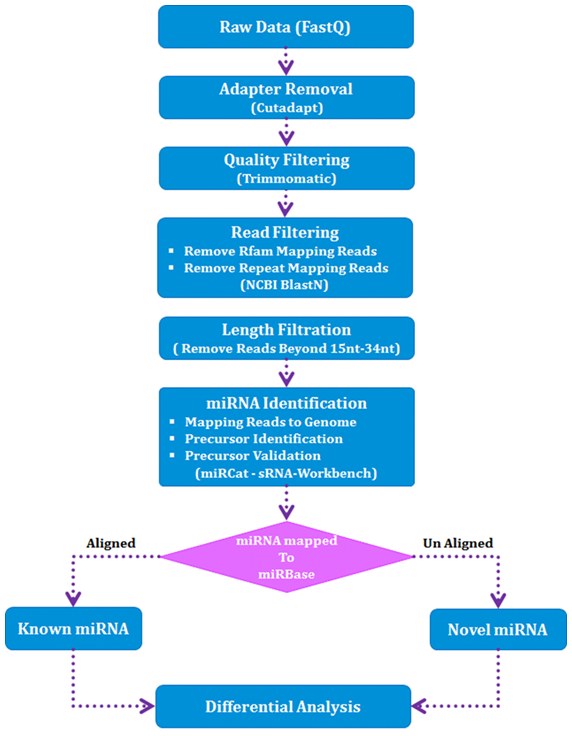

Supplement: S1 Fig — Figure shows bioinformatics work flow of small RNA sequencing and data analysis. (DOCX) [file pone.0233076.s001.docx]
