# Supplementary material for: Genome-wide identification and characterization of microRNAs by small RNA sequencing for low nitrogen stress in potato
Source: PLoS One. 2020 May 19;15(5):e0233076. doi: 10.1371/journal.pone.0233076 (PMC7237020; doi:10.1371/journal.pone.0233076)
Supplement: S2 Fig — miRNAs families distribution for samples: a. KJ_HN_Root; b. KJ_LN_Root; c. KJ_HN_Shoot; d. KJ_LN_Shoot. (DOCX) [file pone.0233076.s002.docx]

**Figure S2. Abundance of reads mapped to Rfam (Family Distribution)**

1. **
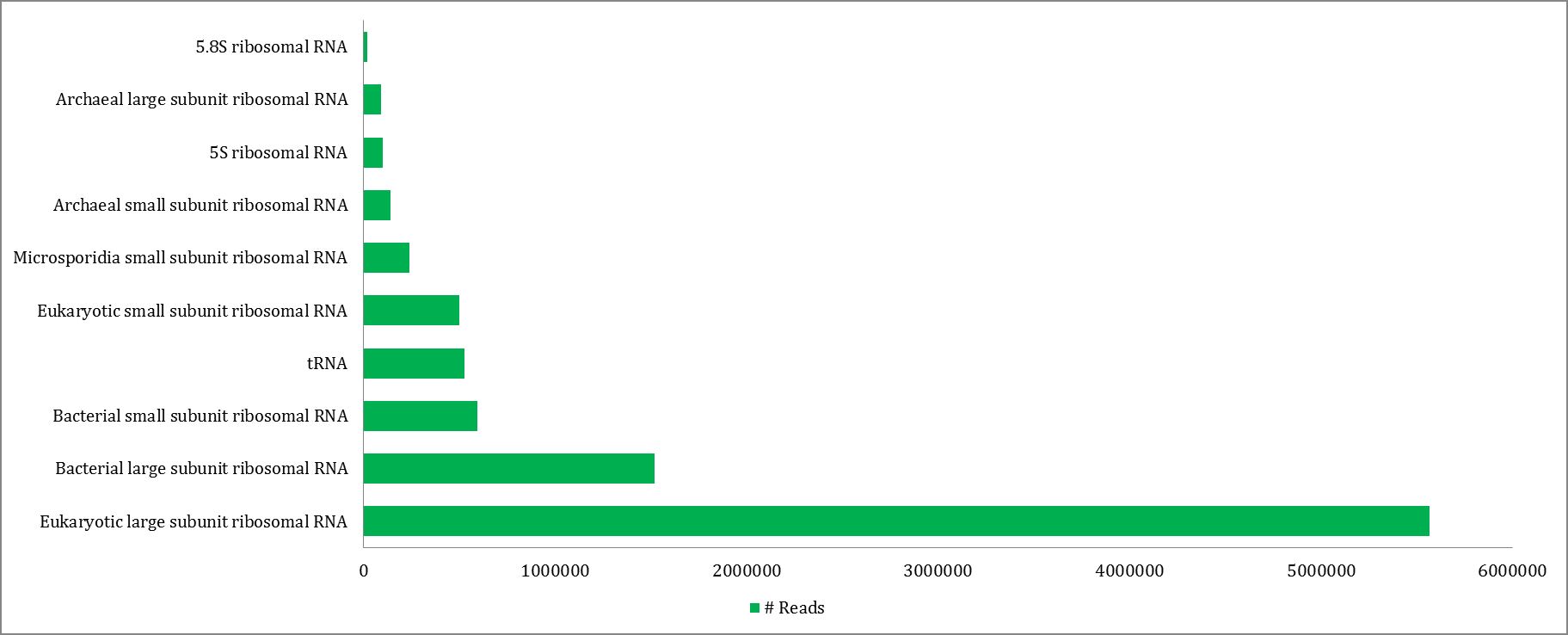
KJ_HN_Root**
2.
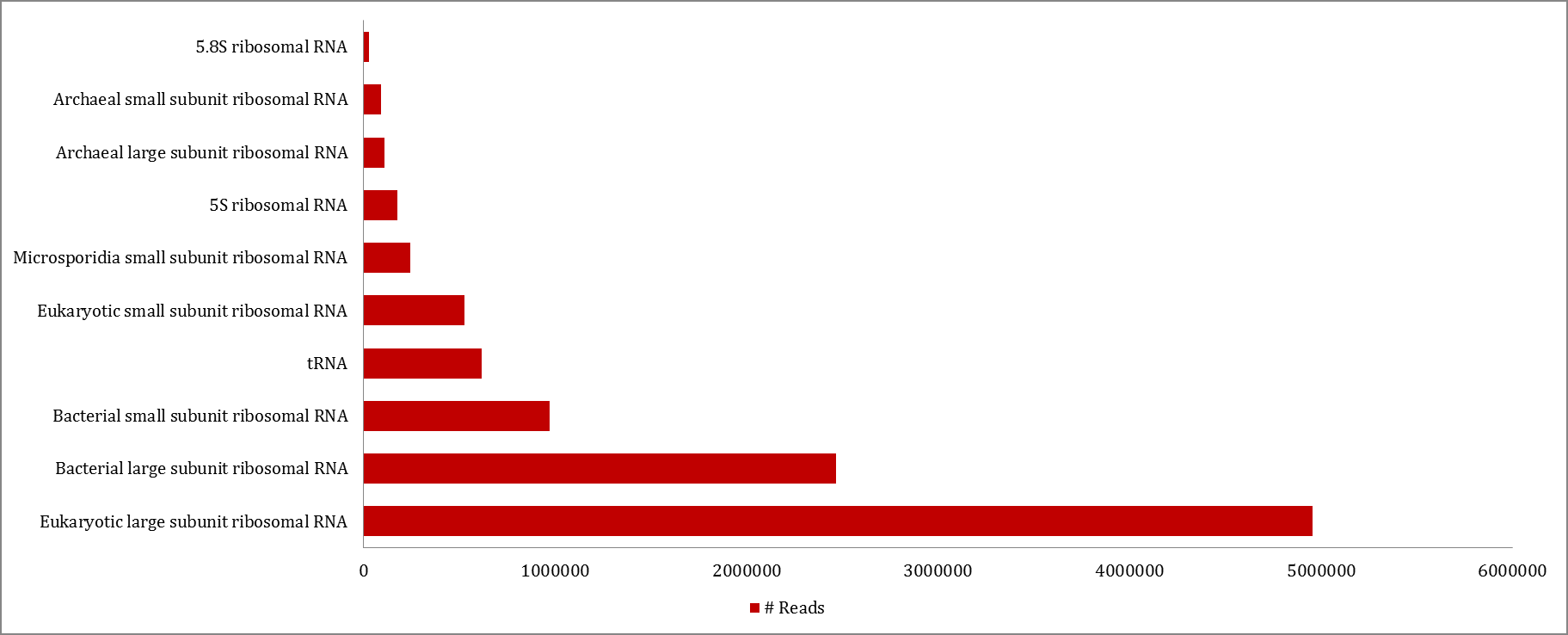
**KJ_LN_Root**
3. **KJ_HN_Shoot**


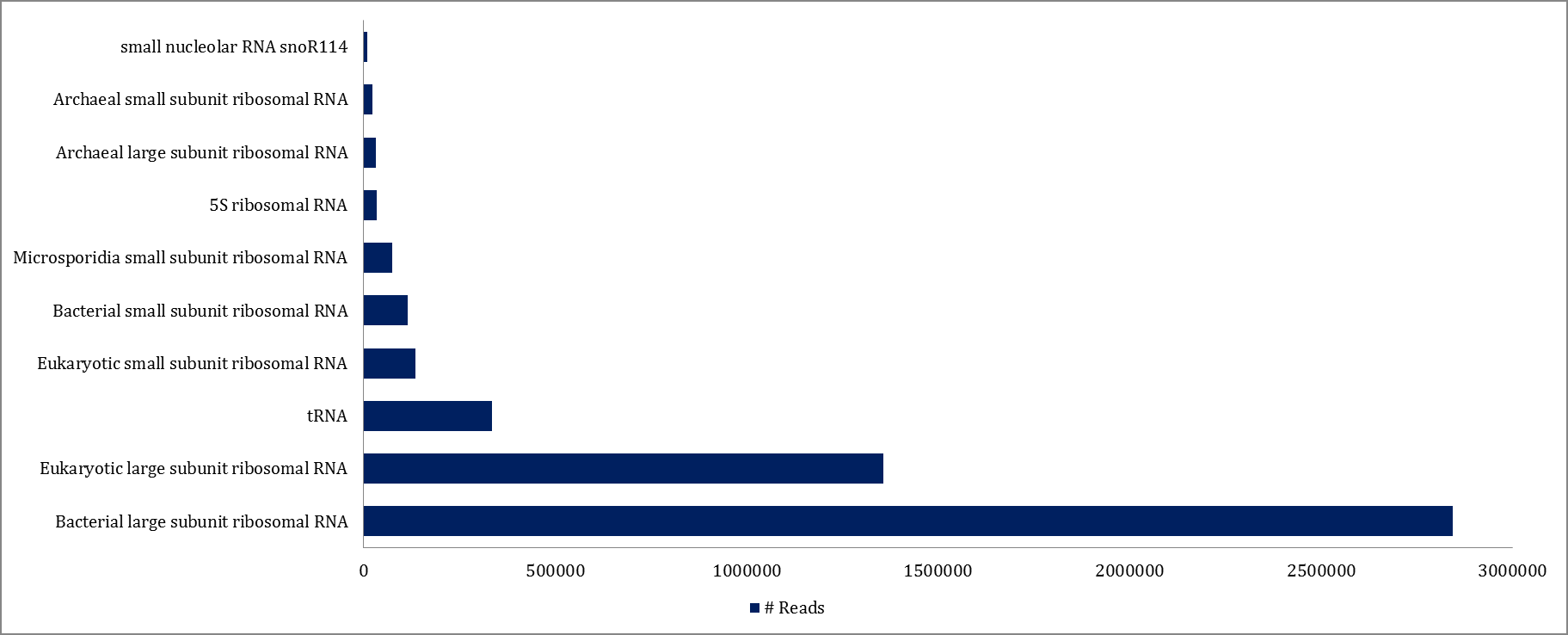


1. **KJ_LN_Shoot**

**
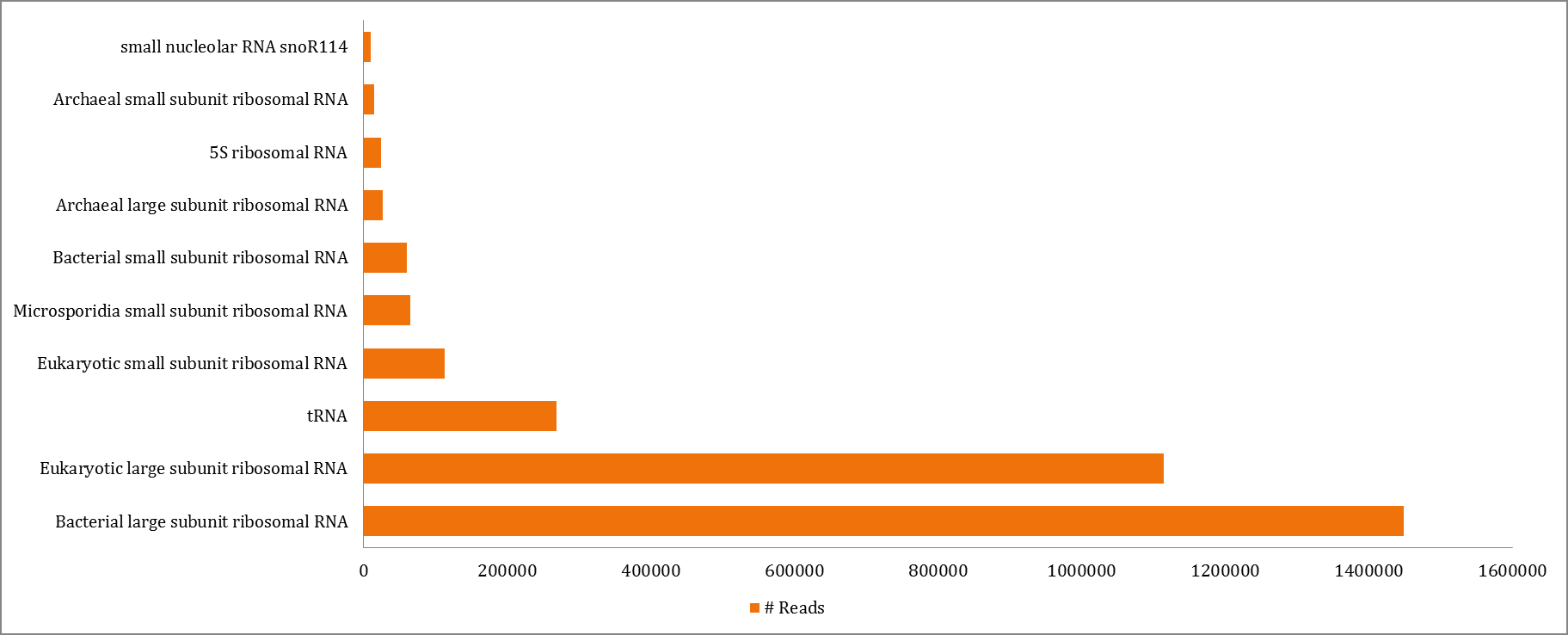
**
