# Supplementary material for: Genome-wide identification and characterization of microRNAs by small RNA sequencing for low nitrogen stress in potato
Source: PLoS One. 2020 May 19;15(5):e0233076. doi: 10.1371/journal.pone.0233076 (PMC7237020; doi:10.1371/journal.pone.0233076)
Supplement: S1 File — Illumina nextseq small RNA library preparation, adapter trimming analysis, quality filtering analysis, uniquification of read tags, reads filtering (removal of known non-coding RNA mapping reads), reads filtering (removal of repeat database mapping reads), reads length filtration, reads mapping to the reference genome, known and novel miRNAs indemnification analysis, differential analysis, precursor sequences secondary structures, miRNAs targets identification analysis. (DOCX) [file pone.0233076.s003.docx]

## Method S1:

## Illumina NextSeq Small RNA library preparation

The small RNA sequencing libraries were prepared from the isolated QC passed total RNA using illumina TruSeq Small RNA Library Preparation Kit as per the manufacturer’s instruction. The protocol includes adapter ligation, reverse transcription, PCR amplification, and pooled gel purification to generate a library product. The RNA 3' adapter is specifically modified to target microRNAs and other small RNAs that have a 3' hydroxyl group resulting from enzymatic cleavage by Dicer or other RNA processing enzymes. The adapters are ligated to each end of the RNA molecule and a reverse transcription reaction is used to create single stranded cDNA. The cDNA is then PCR amplified using a common primer and a primer containing one of index sequences. The amplified PCR product with index sequences were size selected and purified on 6% TBE gel.

**Adapter Trimming Analysis**

Raw reads were screened for adapter contamination using the well-known tool Cutadapt (v 1.16). Any adapter contaminated read meeting the below listed criteria are trimmed off to retain only putative microRNA read population.

| Tool | Cutadapt (v 1.16) |
| --- | --- |
| Citation | *Martin, Marcel. "Cutadapt removes adapter sequences from high-throughput sequencing reads." EMBnet. journal 17.1 (2011): pp-10.* |

- Phred quality score of 20 (i.e. QV 20) has been used to trim terminal low quality bases
- Minimum overlap between adapter and read: 5bp
- Reads with the ambiguous base (i.e. N) has been discarded
- Reads having length below 15bp has been discarded
- InDels are not allowed while aligning adapter sequences to read while detecting contamination

#

# Quality Filtering Analysis

After removing the adapter sequences we have performed the quality filtering analysis to remove any low quality reads or trim the terminal low quality bases. And the analysis has been carried out using the Trimmomatic (v 0.38) in single end mode with minimum read length 15bp and quality cut-off of 20 wherever applicable,

| **Tool** | Trimmomatic. (v 0.38) |
| --- | --- |
| **Citation** | *Bolger, Anthony M., Marc Lohse, and Bjoern Usadel. "Trimmomatic: a flexible trimmer for Illumina sequence data." Bioinformatics 30.15 (2014): 2114-2120.* |

**Uniquification of Read Tags**

Adapter trimmed and quality filtered cleaned reads are formatted into a non-redundant fasta format. Occurrences of each unique sequence reads were counted as sequence tags. Number of reads for each unique tag reflects relative expression level of that miRNA. Considering tags instead of reads allows a high loss-less compression of the original data and simplifies the computational analysis.

**Read Filtering (Removal of Known Non-Coding RNA Mapping Reads)**

Small RNA sequencing data is known to include reads from other category of small noncoding RNAs such as tRNA, snoRNA, snRNA and piRNA etc. Sample wise read tags were blasted against the customized Rfam database of Rfam (Database built after removing miRNA and their precursor sequences from the Rfam 13 build). Parameters used during the Blast analysis were listed below,

| **Tool** | NCBI-Blast (v 2.6.0) |
| --- | --- |
| **Database** | Rfam (v13) |
| **No. of RNA Families** | 2289 (529 families belonging to miRNA and its precursors has been removed) |
| **Citation** | *Kalvari, Ioanna, et al. "Rfam 13.0: shifting to a genome-centric resource for non-coding RNA families." Nucleic acids research 46.D1 (2017): D335-D342.* |

- Blastn variant: blastn-short
- Word size: 7bp
- Low complexity filtering: Off
- Identity: 100%
- Query Coverage: 100%
- Mismatches and Gaps: Not allowed

Any read tag mapped to Rfam database and satisfying the above mentioned criteria’s, those reads were filtered out from the analysis.

**Read Filtering (Removal of Repeat Database Mapping Reads)**

Read tags which were not mapped to Rfam, were mapped to known repeat sequences i.e. Repbase (v 22) using NCBI Blast with below mentioned parameters.

- Word size: 7bp
- Low complexity filtering: Off
- Identity: 100%
- Query Coverage: 100%
- Mismatches and Gaps: Not allowed

Any Tags Matched with above mentioned stringent criteria has been excluded from further analysis.

#

# Read Length Filtration

Reads having length more than 34nt were excluded from the analysis.

Adapter trimmed, quality filtered and length filtered reads were considered as cleaned putative miRNA population, taken further for known and novel miRNA identification.

**Read Mapping to the Reference Genome**

Sample wise length filtered reads were mapped to the Potato reference genome [PGSC v4.04], Precursor sequences were identified by the tool miRCat The UEA small RNA workbench (v 3.2). miRcat analyses the read mapping pattern along with few upstream and downstream bases. It uses the RNAfold to estimate the precursor stability by analyzing the MFE (minimum free energy). Only miRNAs observed on stable precursor sequences were reported. Parameters were used during the analysis were listed below,

| **Tool** | The UEA small RNA workbench (v 3.2) |
| --- | --- |
| **Citation** | 1. *Mohorianu, Irina, et al. "The UEA small RNA workbench: a suite of computational tools for small RNA analysis." MicroRNA Detection and Target Identification. Humana Press, New York, NY, 2017. 193-224.* 2. *Lorenz, Ronny, et al. "ViennaRNA Package 2.0." Algorithms for Molecular Biology 6.1 (2011): 26.* |

- Minimum Abundance: 5 in any one of the sample
- Minimum free energy: -25.0 kCal/mol
- Maximum Gaps: 3bp
- Maximum Genome Hits: 50
- Minimum GC: 20%
- p Value: 0.05

# Known and Novel miRNA Indemnification Analysis

On identifying mature sequences, tool itself compares the candidate mature sequence with miRBase (A known miRNA database) and assigns the miR family id. So based on the assigned miRBase id, we have segregated the miRNAs as known and novel miRNAs.

# Differential Analysis

After aligning onto the respective genome read counts were used for expression profiling for known and novel miRNAs together. miRNA with at least of 10 read support in any of the 4 sample were considered for the differential analysis. TPM (Transcripts Per Million) normalization applied to normalize the read count to the length of the miRNA population per sample. TPM values were then log (base 2) transformed to get Fold Expression values. And finally fold change values are calculated by subtracting respective fold expression values. Fold Change value of above zero is considered as Up-Regulated whereas below zero is considered as Down-Regulated.

**Precursor Sequences Secondary Structures**

In-house custom wrapper scripts based on RNAfold was used to generate the precursor sequence secondary structure. We also highlighted respective mature miRNA and mature* wherever available. These images will largely help in visually filtering false positives based on secondary structure principles like number of bulges/gaps etc.

**miRNA Target Identification Analysis**

microRNAs targets the coding sequences of the genome. We used psRNATarget finder to predict the potential targets (Obtained from Group Phureja DM1-3 516R44 (CIP801092) Genome 3.4 transcripts) per microRNAs. Tool identifies target transcripts through the following mechanism,

- Analyzing complementary matching between sRNA and target using
- Evaluating target site accessibility by calculating unpaired energy (UPE)
- We have used all default parameters as latest version of the tool (v 2017) claimed to have pre optimized default scoring schema.

Some of the key parameters used during the analysis are,

- - Penalty for mismatches (Including G:U pair): 1
  - Extra weight in seed region: 1.5
  - Mismatches allowed in seed region: 1 (Bulges on targets are allowed)
  - Gap opening penalty: 2
  - Gap Extension penalty: 0.5
  - Seed region: 2-13 nucleotides
  - HSP Size: 19 bases

34,135 unique transcript sequences were targeted by 723 unique miRNA sequences (including known and novel microRNAs). In-Silico target finding analysis it is evident that, transcript expression was regulated by cleavage mechanism.
