# Supplementary material for: Genome-wide identification and characterization of microRNAs by small RNA sequencing for low nitrogen stress in potato
Source: PLoS One. 2020 May 19;15(5):e0233076. doi: 10.1371/journal.pone.0233076 (PMC7237020; doi:10.1371/journal.pone.0233076)
Supplement: S1 Table — a) raw reads data statistics, b) adapter trimming analysis summary statistics, c) quality trimming analysis summary statistics, d) read tags uniquification summary statistics, e) Rfam database mapped read filtering statistics, f) repeat database mapped read filtering statistics, and g) read length filtration summary analysis. (DOCX) [file pone.0233076.s004.docx]

**Table S1. Details of data generation by small RNA sequencing for N stress in potato**

| **a) Raw Data Statistics** | | | | |  | | | | | | |
| --- | --- | --- | --- | --- | --- | --- | --- | --- | --- | --- | --- |
| Sample ID | | | | | # Single End Reads | | | | | | |
| KJ_HN_Root | | | | | 21,929,322 | | | | | | |
| KJ_LN_Root | | | | | 21,693,480 | | | | | | |
| KJ_HN_Shoot | | | | | 14,873,782 | | | | | | |
| KJ_LN_Shoot | | | | | 11,955,717 | | | | | | |
| **b) Adapter Trimming Analysis Summary Stats** | | | | | | | | | | | |
| Description | | | | KJ_HN Root | KJ_LN Root | | | KJ_HN Shoot | | | KJ_LN Shoot |
| Total Reads | | | | 21,929,322 | 21,693,480 | | | 14,873,782 | | | 11,955,717 |
| Reads With Adapters | | | | 21,738,147 | 21,507,021 | | | 14,743,995 | | | 11,850,183 |
| Read Length <15nt | | | | 2,772,604 | 3,321,560 | | | 2,269,815 | | | 2,828,898 |
| Adapter Trimmed Reads | | | | 18,959,902 | 18,179,907 | | | 12,471,477 | | | 9,018,624 |
| **c) Quality Trimming Analysis Summary Stats** | | | | | | | | | | | |
| Sample ID | Total Reads | | | | | High Quality Reads | | | | Low Quality Reads | |
| KJ_HN_Root | 18,959,902 | | | | | 18,726,141 (98.77%) | | | | 233,761 | |
| KJ_LN_Root | 18,179,907 | | | | | 17,955,245 (98.76%) | | | | 224,662 | |
| KJ_HN_Shoot | 12,471,477 | | | | | 12,223,275 (98.01%) | | | | 248,202 | |
| KJ_LN_Shoot | 9,018,624 | | | | | 8,913,648 (98.84%) | | | | 104,976 | |
| **d) Read Tag Uniquification Summary Stats** | | | | | | | | | | | |
| Sample ID | | High Quality Reads | | | | | Unique Tags | | Average Read Length | | |
| KJ_HN_Root | | 18,726,141 | | | | | 3,990,938 | | 24.3 nt | | |
| KJ_LN_Root | | 17,955,245 | | | | | 4,516,972 | | 25.3 nt | | |
| KJ_HN_Shoot | | 12,223,275 | | | | | 3,010,351 | | 23.7 nt | | |
| KJ_LN_Shoot | | 8,913,648 | | | | | 2,650,649 | | 23.5 nt | | |
| **e) Rfam database mapped read filtering stats** | | | | | | | | | | | |
|  | | | KJ_HN Root | | KJ_LN Root | | | KJ_HN Shoot | | | KJ_LN Shoot |
| # Reads | | | 18,726,141 | | 17,955,245 | | | 12,223,275 | | | 8,913,648 |
| # Tags | | | 3,990,938 | | 4,516,972 | | | 3,010,351 | | | 2,650,649 |
| # Reads Mapped | | | 9,440,070 | | 10,329,887 | | | 5,036,970 | | | 3,220,323 |
| # Tags Mapped | | | 795,022 | | 1,166,230 | | | 197,470 | | | 176,387 |
| # Reads Unmapped | | | 9,286,071 | | 7,625,358 | | | 7,186,305 | | | 5,693,325 |
| # Tags Unmapped | | | 3,195,916 | | 3,350,742 | | | 2,812,881 | | | 2,474,262 |
| **f) Repeat database mapped read filtering stats** | | | | | | | | | | | |
|  | | | | KJ_HN Root | KJ_LN Root | | | KJ_HN Shoot | | | KJ_LN Shoot |
| # Reads | | | | 9,286,071 | 7,625,358 | | | 7,186,305 | | | 5,693,325 |
| # Tags | | | | 3,195,916 | 3,350,742 | | | 2,812,881 | | | 2,474,262 |
| # Reads Mapped | | | | 108,788 | 86,606 | | | 128,322 | | | 112,266 |
| # Tags Mapped | | | | 38,594 | 35,859 | | | 40,628 | | | 38,875 |
| # Reads Unmapped | | | | 9,177,283 | 7,538,752 | | | 7,057,983 | | | 5,581,059 |
| # Tags Unmapped | | | | 3,157,322 | 3,314,883 | | | 2,772,253 | | | 2,435,387 |
| **g) Read Length Filtration Summary Analysis** | | | | | | | | | | | |
|  | | | | KJ_HN Root | KJ_LN Root | | | KJ_HN Shoot | | | KJ_LN Shoot |
| # Reads | | | | 9,177,283 | 7,538,752 | | | 7,057,983 | | | 5,581,059 |
| # Tags | | | | 3,157,322 | 3,314,883 | | | 2,772,253 | | | 2,435,387 |
| # Reads (15nt -34nt) | | | | 8,710,979 | 6,820,194 | | | 6,904,591 | | | 5,464,795 |
| # Tags (15nt -34nt) | | | | 2,995,557 | 2,980,953 | | | 2,730,537 | | | 2,414,279 |
