# Supplementary material for: Genome-wide identification and characterization of microRNAs by small RNA sequencing for low nitrogen stress in potato
Source: PLoS One. 2020 May 19;15(5):e0233076. doi: 10.1371/journal.pone.0233076 (PMC7237020; doi:10.1371/journal.pone.0233076)
Supplement: S14 Table — Summary of RT-qPCR primers used for gene expression analysis of miRNAs and their corresponding targets. (DOCX) [file pone.0233076.s017.docx]

**Table S14.** Validation of selected miRNAs and their predicted targets by RT-qPCR analysis.

| SN | Primer sequence of target gene  (5’🡪3’) | Target gene ID | Gene name | miRNA  (miRBase ID) | Location of miRNA on potato chromosome # | Gene expression value (Log_2_ fold change) of RT-qPCR of | |
| --- | --- | --- | --- | --- | --- | --- | --- |
|  |  |  |  |  |  | miRNA | Target gene |
| Conserved miRNAs (root) | |  |  |  |  |  |  |
| 1. | F: GAGGAGCAGTGAAGAGGAATTT  R: CTGCCATCTAGTGCTGATAAGG | PGSC0003DMT400045253 | Universal stress protein family protein | stu-known-mir34  (miR482) | 4 | 1.68±0.26 | -1.72±0.17 |
| 2. | F: GGCGGAGCTACACAGTTAAT  R: GCCTGAAACAGAGCAGGTATAA | PGSC0003DMT400043794 | Nitrate transporter | stu-known-mir52  (miR156miR157) | 2 | 1.59±0.22 | -1.56±0.36 |
| 3. | F: CATCAACGCTGCACTCAATAAC  R: CGTGACATAGGGATCAGTGTAAG | PGSC0003DMT400001824 | Laccase | stu-known-mir13  (mir397) | 7 | -2.42±0.16 | 2.50±0.29 |
| 4. | F: TGTCCGTTCTCTGTGGTTTAC  R: GCTGTCATGTCCTGCACTAT | PGSC0003DMT400040827 | Calmodulin-binding heat-shock protein | stu-known-mir24  (miR398) | 11 | -4.02±0.19 | 4.10±0.47 |
| 5. | F: GCTGCAGCTTTGATGAGTTTAG  R: TCTCCCAGCCTGACGTATAG | PGSC0003DMT400022057 | Heat shock protein binding protein | stu-known-mir46  (miR398) | 12 | -5.21±0.21 | 5.27±0.28 |
| Conserved miRNAs (shoot) | |  |  |  |  |  |  |
| 6. | F: TGGACTACCTATGCCCAAATG  R: GGCGGGATTTCAAAGGATTTAC | PGSC0003DMT400032493 | Nitrate transporter | stu-known-mir57  (miR319) | 3 | 1.85±0.36 | -1.91±0.20 |
| 7. | F: TGGAAATAGTGTGGAGGACAAC  R: ACCCATAGCAAACACCTGATAG | PGSC0003DMT400039292 | Amino acid transporter | stu-known-mir37  (miR156) | 7 | 1.82±0.15 | -1.88±0.14 |
| 8. | F: GAGGGAGTTAGGCAACATAACA  R: AGCTTACAGCCGGATAATGAC | PGSC0003DMT400026824 | Serine-threonine protein kinase, plant-type | stu-known-mir61  (miR398) | 3 | -1.07±0.16 | 1.10±0.17 |
| 9. | F: GAGATACCAGCACACATCCATC  R: CTGGAGAATCCGACACGAATAC | PGSC0003DMT400006972 | F-box family protein | stu-known-mir14  (miR5303) | 1 | -1.21±0.21 | 1.30±0.25 |
| Putative novel miRNAs (root) | |  |  |  |  |  |  |
| 10. | F: GTTGAGGAGAAAGAGCAGAAGA  R: AGATTCGCAGTCCACTTTGT | PGSC0003DMT400012426 | Transcription factor | stu-novel-mir963  (NA) | 12 | 2.89±0.38 | -2.95±0.15 |
| 11. | F: CAACCAGAGACGGCCTTAAA  R: TCTAGTCCAATACCTCCGACAA | PGSC0003DMT400060216 | Zinc finger protein | stu-novel-mir712  (NA) | 4 | -2.47±0.36 | 2.50±0.21 |
| Putative novel miRNAs (shoot) | |  |  |  |  |  |  |
| 12. | F: TCGATGAATGGCTACCAATAACA  R: GCGTAAGGGAGACTAAGAACAC | PGSC0003DMT400044953 | Amino acid transporter | stu-novel-mir1052  (NA) | 12 | 2.61±0.22 | -2.70±0.20 |
| 13. | F: TGTCTATACTGCCGAGGTATCC  R: TGGTGTGGTAAACCAGCTAATG | PGSC0003DMT400011287 | Sugar transporter | stu-novel-mir858  (NA) | 12 | -1.54±0.17 | 1.62±0.36 |

NA: Not Available
