# Supplementary material for: Genome-wide identification and characterization of microRNAs by small RNA sequencing for low nitrogen stress in potato
Source: PLoS One. 2020 May 19;15(5):e0233076. doi: 10.1371/journal.pone.0233076 (PMC7237020; doi:10.1371/journal.pone.0233076)
Supplement: S15 Table — Primer sequences of 5’ RLM-RACE used for target validation and sequences of cloned product of RACE product. (DOCX) [file pone.0233076.s018.docx]

**Table S15:** Gene-specific primers (5’ and 3’) designed for RLM-RACE amplification and sequences of RACE product cloned

1. PGSC0003DMT400001824 (Laccase): Target of miR397

| Type | Sequence | Start | Length | Tm | GC Percent |
| --- | --- | --- | --- | --- | --- |
| Forward Primer | CCGATCCGACCCGGACATAGCTACAC | 316 | 26 | 70.148 | 61.538 |
| Reverse Primer | CCCATCCTCCAACTGGAACGCCAAT | 1558 | 25 | 70.041 | 56 |

>PGSC0003DMC400001345 PGSC0003DMT400001824

ATGAGGCATCAAATTTTCTTGTTATTTGCTTGCACCACAATAGCTCTTTTAGCTTCTTGT

TCTTCATTGGTTTCAGCTGAAGTTGTTGAGCATTCTTTTCATGTGCAAAACCGTACTATA

ACGAGATTGTGCCGTAGACAAGTAATTACTGCTGTAAATGGAAGTCTTCCTGGTCCAACC

ATACGTGTAAAAGAAGGAGACACCCTTGTGGTTCATGTCTACAACCTTTCACCTTACAAT

CTCACTATTCATTGGCATGGAGTTTTCCAGCTATTAAGTGGGTGGGCTGATGGACCGGAG

TTTGCGACCCAGTGCCCGATCCGACCCGGACATAGCTACACATACAAGTTTAAAATAACA

GGACAAGAAGGGACTCTATGGTGGCATGCACACGTGTCATGGCTTAGAGCCACAGTTCAT

GGTGCACTCATTATTCGCCCAAAAAAAGGCCACTCTTTTCCTTTCCCTAAACCTTATAGA

GAATTTCCTATTCTCTTAGGAGAATGGTGGAATGCCAATGTTGTGGATGTGGAGAATGCA

GCGCTAGCAACTGGCTCAGCACCTAATAACTCTAATGCTTACACTATTAACGGCTGGCCG

GGCGATCTTTACCCTTGCTCTGTTAATCAAACGTACAAGTTGAAGGTGAAACACGGAAAA

ACATATCTCTTACGT**ATCATCAACGCTGCACTCAAT**AACCAACTTTTTTTCAAGATCGCC

AATCATAAAATGAAAGTTGTCGCGGTTGATGCTGCTTACACTGATCCCTATGTCACGGAT

GTAGTTGTCACCGGGCCGGGCCAGACGACTGACGTCCTCTTGACGGCCGATCAACTACCG

GCGTCGTACTACGTGACGGCTAACCCCTATGCTAGTGCAGCCGGGGTGCCATTTGACAAC

ACCACAACTAGAGGAATTATAGTGTATGAAGATGCATTACTACCATCAACTCCCATAATG

CCAATTCTACCTGCCTTTAATGACACACCAACAGCCCATAAATTTTTCACTAATATAACT

GGGCTTGTAACTGGCCCATTTTGGAACCCACCACCTCGAAAAGTGGACGAACACATGTTT

ATCACTATTGGGCTGGGCCTAACTGCTTGTGGAAAATCAAGAAATGCAACATGTGGAGGC

CCAAATGGACAACGATTTTCGGCTAGTATGAACAATGCATCTTTCCAGTTTCCAGACAAG

ATTTCAATGTTGGAAGCCTTTTTCTACAATGTTGGTGGAATTTACACTACTGATTTCCCA

GACAAACCACCATTGAAGTTTGACTATACAAACCCAAATAATAGCATGAATCCTGCTATT

ATAATGACCAAAAAATCAACAAAAGTGAAGAAAATTAAGTTCAATTCCACAGTTGAAATT

GTGTTCCAAAATACTGCTTTGATTGGGATAGAAAATCATCCTATTCACTTGCATGGATTC

AATTTCCATGTATTGGCACAAGGCTTTGGCAATTATAACCCTGCAGTCGATAGGAAAAAA

TTCAACTTTGTCAATCCACAAGAACGTAACACAATTGGCGTTCCAGTTGGAGGATGGGCT

GTCATTAGATTTCGAGCTAACAATCCAGGTGTATGGTTGATGCATTGTCATTTGGATGTT

CACTTGCCTTGGGGTTTATCAACAGCTTTTGTTGTTGAAAATGGGCCAACATTATCAACT

ATGCTACCTCCTCCTCCACCAGATCTACCTAAATGCTAG

2. PGSC0003DMT400026824 (Serine-threonine protein kinase, plant-type): Target of miR398

| Type | Sequence | Start | Length | Tm | GC Percent |
| --- | --- | --- | --- | --- | --- |
| Forward Primer | CTGGTCGCGTGCCACCTTGCTTAG | 1643 | 24 | 70.463 | 62.5 |
| Reverse Primer | GGCGGAGATTGCGCAACATCTCACATT | 2551 | 27 | 70.55 | 51.852 |

>PGSC0003DMC400018288 PGSC0003DMT400026824

ATGGGAAGAAGTTGCAATCTTCTCTTTGCACTTGTGGTTTTCATTCTGCTCCATACTTCA

TTCTCTACTGTTCCAAATATTAGTACTGATGAAGCTGCTCTTCTTGCATTAAAATCTCAC

ATTTCTTTTAGTCCTAACAACATCTTAGCAACCAACTGGTCTTCTTCCACCCCTGTTTGC

ACCTGGATTGGAATCACTTGCACCTCCCGACACCATCGAGTCACAGCTTTAGACATTTCT

AGTATGCAACTTCATGGTACCATTCCTCCACACCTTGGAAACCTCTCATTTCTTGTTTCC

CTCAATATTAATAACAACAGATTCCATGGGAATTTACCCCAAGAGTTGGCTCATTTGCAG

AGGTTGAAATTGATTGATGTCACAAGTAATAACTTAACTGGTGCCATTCCAACATCTTTA

GGTTTGCTAGTAGACCTTCGCATTTTGCGCCTGTCTAGCAATCAATTTTCCGGAAAAATC

CCATCTTCCCTTTCCAATCTAACAAAGCTGGAAGTGTTGAGAATAACGGGAAATTTTCTC

GAAGGAGAGATCCCTCGAGAACTTGGTGATCTTCATTACATGACTGCTTTAAATCTGGAA

AGTAACCATCTTACTGGCTCTATACCACCATCAATTTATAACATTACAACCATGCGAATC

ATTGGTCTTACCAACAATAATCTTACTGGTGAGCTTCCAACAACTATATGTGACTATCTT

CCAAACTTGGAAGGGCTTCACCTCTCAACAAACATCCTAGGTGGCATTATTCCACCAAAC

TTGGAAAAATGCAAAAAGCTCAAAGTCTTGTCATTGTCTGTCAATGAGTTCACTGGAACT

ATACCGAGGGAGTTAGGCAACATAACAAATCTTGCAGAATTATACCTTGGATTGCTGCAC

TTCGAAGGAGAGATACCAGTGGAGCTAGGTAATCTTAAGAAACTACAGATTCTGTCATTA

TCCGGCTGTAAGCTTACTGGTTCCATCCCCACAAGCATTTTCAACATGTCAGCGCTGCGG

GCAGTCGTACTTGACGAAAACATGCTTTCAGGTAATCTACCAGCAGATTTAGGCAGTGGG

ATCCCCAGCCTAGAAACATTGTTTTGTGCAAACAACTATCTGAGTGGATTTATATCTGCT

ACTATCTCAAATGCTTCAAGACTCAGTGAGATTGATCTTTCAATCAATAGTTTCACAGGT

CCAATTCCTCAATCACTTGGTAACTTAGAAAACCTTGAGGTTTTGGACCTGCAGGTGAAC

AATTTTGTCAGCGATTCAGCATTGAGCTTCCTGACATCTTTGACACACTGTAGGAAACTG

AGAGAACTCACCTTTGCTAAGAATCCCTTGGAAGGTTTTTTCCCTGCATCAATTGGAAAT

TTCTCTGATTTTCTGCAATTTTTCGAAGGATGGACTTGTAAACTGAAAGGCTCCATTCCT

GGAGAAATTGGTAATCTTACCGGTGTGATAAGGATAAATTTTTCACAAAATGATTTGACT

GGACATATTCCAAAAACTATCCAAGGCATGCTGAACCTTCAAGAATTTTACCTAGAGAGC

AACAAGATAGAAGGAGTCATACCAGATTCTATCTGCAATTTAAAGAATCTTGGAGCATTA

TTCTTGTCAGGTAATCAATTTTCTGGTCGCGTGCCACCTTGCTTAGGAAAAGTTACCAGT

ATGAGATATCTTTCTCTAGCTGATAACATGCTTAATTCGAGTTTACCCGAAAGCTTGGGG

AGCCTTCCTGATCTCATAGAGTTCAATTTTTCATCCAATTTATTGCATGGCAAAATTCCT

ATTGAGATTGGAAATTTAAAGTCTGCAACACTCATTGATCTGTCAAAAAATGATTTTTTT

GGTATGATCCCTAGCACTCTAGAGGGTCTGGATAGATTGATTAGTCTTTCTCTAGCACAT

AATAAATTAGATGGGCCTATTCCAGATTCATTTAGAAAAATACTGGCCTTGGAATACTTG

GATTTGTCCAGTAACAATCTTAGTGGTGAAATCCCAAAGTCATTAGAAGCTCTTGTGTAT

CTCAAATACTTGAACTTCTCATTTAATGAATTCAGTGGAGAAATTCCCACGGGTGGTCCT

TTTGCAAATGCCACAGATCAATCTTTCTTGTCCAATTATGGGCTCTGTGGTGATTCTAAG

TTTCATGTTTCACCATGCGTCATCAAATCTCCCAAGAGGTCAAAGAAAAAAAAGGCAATT

TTGGTTTTGTACATCCTTTTGGGAGTAGGTATGCTATTTCTTGCATTAGCCGTCACATAT

GTATTTTTGAGATTGAGAAAGAAAAAAAAGAATGCAGGTCAAGCTGATGTGTCTCTGATA

AAATGTCATGAAAGAATTTCCTATTATGAACTTGAACAAGCAACCGAAGGATTCAATGAA

AGCAACTTGCTTGGTAACGGGAGTTTCAGCAAGGTCTACAAAGGGATACTTAAGGATGGT

ACTCTTTTGGCTTCAAAGGTATTCAATGTGCAATTGGAGGGTGCATTCAAAAGTTTTGAT

ACAGAATGTGAGATGTTGCGCAATCTCCGCCACCGAAATCTGACCAAAGTCATCACCAGT

TGCTCCAACCTTGATTTCAAAGCCTTAGTGTTGGAATACATGTCCAATGGGACACTTGAT

AAATGGCTATACTCTCACAACTTGTTCTTGGATTTATTTCACAGATTAGATATAATGATA

GATGTTGCATCTGCAATGGTCTATCTCCACAGTGGTTGTTCAAATCCTGTGGTGCATTGT

GACTTAAAGCCAAGCAATGTCTTGCTAGATCAAGAAATGGTTGGCCATGTCAGTGATTTT

GGCATTGCAAAATTGTTAGGTGCAGGGGAGACTTTTGTTCAAACAAGGACAATAGCAACC

ATTGGATATATTGCTCCAGAGTATGGACAAGATGGAATAGTATCCACGAGCTGTGATGTT

TATAGTTTCGGCATCCTGATGATGGAGACATTTACAAGAATCAGACCAGGTGATGAAAGA

TTT**ACTGGTGACTTGAGCATACGA**CGTTGGGTAAGCGATTCTTTTCCAGATGAGATTCAT

AAGGTGGTGGATGCTAATTTGGTACAGCTAGAGGATGAACGAATTGACGCAAAGATGCAG

TGTCTGTTGTCTATCATAGAGTTAGCTTTGAGCTGCACTTTAGTGACACCTGATGCAAGA

ATTAGTATGGAAGATTCTCTTTCAACACTTCAAAAGATCAGGCTCCTGTTTGTCAATAGT

CGCCGCTAG

**Sequences of RACE product cloned**

**1. miR397: ATTGAGTGCAGCGTTGATGAC**

| miRNA_Acc. | miRBase ID | Mature_miRNA | Target_Acc. | Target_Desc. | Expectation | UPE | miRNA_start | miRNA_end | Target_start | Target_end | miRNA_aligned_fragment (5’-3’) | Target_aligned_fragment (5’-3’) | Inhibition |
| --- | --- | --- | --- | --- | --- | --- | --- | --- | --- | --- | --- | --- | --- |
| stu-known-mir13 | mir397 | ATTGAGTGCAGCGTTGATGAC | PGSC0003DMT400001824 | Laccase | 0 | 7.687 | 1 | 21 | 713 | 733 | AUUGAGUGCAGCGUUGAUGAC | AUCAUCAACGCUGCACUCAAU | Cleavage |

>PGSC0003DMT400001824_Laccase 5’- AUCAUCAACGCUGCACUCAAU -3’

.::::::::::::::::::::

>miR397 3’- CAGUAGUUGCGACGUGAGUUA -5’

>Laccase1 (forward primer)

TTGGCTGCAGGCATGCTATACAGGACAGAAGGGACTCTATGGTGGCATGCACACGTGTCATGGCTTAGAGCCACAGTTCA

TGGTGCACTCATTATTCGCCCAAAAAAAGGCCACTCTTTTCCTTTCCCTAAACCTTATAGAGAAGTTCCTATTCTCTTAG

GAGAATGGTGGAATGCCAATGTTGTGGATGTGGAGAATGCAGCGCTAGCAACTGGCTCAGCACCTAATAACTCTAATGCT

TACACTATTAACGGCTGGCCGGGCGATCTTTACCCTTGCTCTGTTAATCAAACATACAAGTTGAAGGTGAAACACGGAAA

AACATATCTCTTACGTATCATCAACGCTGCACTCAATAACCAACTTTTTTTCAAGATCGCCAATCATAAAATGAAAGTTG

TCGCGGTTGATGCTGCTTACACTGATCCCTACGTCACGGATGTAGTTGTCACCGGGCCGGGCCAAACGACTGACGTCCTC

TTGACGGCCGATCAACTACCGGCGTCGTACTACATGACGGCTAACCCCTATGCTAGTGCAGCCGGGGTGCCATTTGACAA

CACCACAACTAGAGGAATTATAGTGTATGAAGATGCATTACTACCATCAACTCCCATAATGCCAATTCTACCTGCCTTTA

ATGACACACCAACAGCCCATAAATTTTTCACTAATATAACTGGGCTTGTAACTGGGCCCATTTTGGAACCCACCACCTCG

AAAAGTGGACGAACACATGTTTATCACTATTGGGCTGGGGCCTAACTGCTTGTGGAAAATCAAGAAATGCACATGTGGAG

GCCCAAATGGACAACGATTTTCGGCTAGTATGAACAATGCATCTTTCCAGGTTCCAGACAGATTCAATGGTGGAAGCCTT

TTTTCTACATGTGTGATTACACTACTGATTCCAAGACCAACCCACCCCTTTG

>Laccase2 (forward primer)

ACGGCTCGACGCATGCTATACAGGACAGAAGGGACTCTATGGTGGCATGCACACGTGTCATGGCTTAGAGCCACAGTTCA

TGGTGCACTCATTATTCGCCCAAAAAAAGGCCACTCTTTTCCTTTCCCTAAACCTTATAGAGAAGTTCCTATTCTCTTAG

GAGAATGGTGGAATGCCAATGTTGTGGATGTGGAGAATGCAGCGCTAGCAACTGGCTCAGCACCTAATAACTCTAATGCT

TACACTATTAACGGCTGGCCGGGCGATCTTTACCCTTGCTCTGTTAATCAAACATACAAGTTGAAGGTGAAACACGGAAA

AACATATCTCTTACGTATCATCAACGCTGCACTCAATAACCAACTTTTTTTCAAGATCGCCAATCATAAAATGAAAGTTG

TCGCGGTTGATGCTGCTTACACTGATCCCTACGTCACGGATGTAGTTGTCACCGGGCCGGGCCAAACGACTGACGTCCTC

TTGACGGCCGATCAACTACCGGCGTCGTACTACATGACGGCTAACCCCTATGCTAGTGCAGCCGGGGTGCCATTTGACAA

CACCACAACTAGAGGAATTATAGTGTATGAAGATGCATTACTACCATCAACTCCCATAATGCCAATTCTACCTGCCTTTA

ATGACACACCAACAGCCCATAAATTTTTCACTAATATAACTGGGCTTGTAACTGGGCCCATTTTGGAACCCACCACCTCG

AAAAGTGGACGAACACATGTTTATCACTATTGGGCTGGGGCCTTACTGCATGTGGGACATCTAGAGATGCAGATGTGCAG

GCCCAATTGGACGACGAATTACGCCTAGTATCAACAATCGATCATTCGAGCTTCGAGACAGATTCAATGGTGGTAGCCAT

TTTTCTACATGTGTGATTACACTACTGATTCCAAGACCAACCCACCCCTTTG

>Laccase3 (Reverse primer)

GGTAGTCTGTGGATTGATAAGTTGATTTTTTCCTATCGACTGCAGGGTTATAATTGCCAAAGCCTTGTGCCAATACATGG

AAATTGAATCCATGCAAGTGAATAGGATGATTTTCTATCCCAATCAAAGCAGTATTTTGGAACACAATTTCAACTGTGGA

ATTGAACTTAATTTTCTTCACTTTTGTCGATTTTTTGGTCATTATAATAGCAGGATTCATGCTATTATTTGGGTTTGTAT

AGTCAAACTTCAATGGTGGTTTGTCTGGGAAATCAGTAGTGTAAATTCCACCAACATTGTAGAAAAAGGCTTCCAACATT

GAAATCTTGTCTGGAAACTGGAAAGATGCATTGTTCATACTAGCCGAAAATCGTTGTCCATTTGGGCCTCCACATGTTGC

ATTTCTTGATTTTCCACAAGCAGTTAGGCCCAGCCCAATAGTGATAAACATGTGTTCGTCCACTTTTCGAGGTGGTGGGT

TCCAAAATGGGCCAGTTACAAGCCCAGTTATATTAGTGAAAAATTTATGGGCTGTTGGTGTGTCATTAAAGGCAGGTAGA

ATTGGCATTATGGGAGTTGATGGTAGTAATGCATCTTCATACACTATAATTCCTCTAGTTGTGGTGTTGTCAAATGGCAC

CCCGGCTGCACTAGCATAGGGGTTAGCCGTCACGTAGTACGACGCCGGTAGTTGATCGGGCCGTCAAGAGGACGTCAGTC

GTCTGGGCCCGGGCCCGGTGACAACTACATCCGTGATGTAGGGATCAGTGTAAGCAGCATCAACCGCGACACTTTCATTT

ATGATTGGCGATCTTGAAAAAAAGTGTATGAGTGCAGCGTTGATGATACGTAAGAAGAATATGGTTTTCGATGTTTCACG

TCAACTGTATCGTTGATACAGAGCCAAGGGTTAAAGAATTCGCCCGGGCGCACAGC

>Laccase4 (Reverse primer)

GGAGAGTTCTTGTGGATTGACAAGTTGATTTTTTCCTATCGACTGCAGGGTTATAATTGCCAAAGCCTTGTGCCAATACA

TGGAAATTGAATCCATGCAAGTGAATAGGATGATTTTCTATCCCAATCAAAGCAGTATTTTGGAACACAATTTCAACTGT

GGAATTGAACTTAATTTTCTTCACTTTTGTCGATTTTTTCGTCATTATAATAGCAGGATTCATGCTATTATTTGGGTTTG

TATAGTCAAACTTCAATGGTGGTTTGTCTGGGAAATCAGTAGTGTAAATTCCACCAACATTGTAGAAAAAGGCTTCCAAC

ATTGAAATCTTGTCTGGAAACTGGAAAGATGCATTGTTCATACTAGCCGAAAATCGTTGTCCATTTGGGCCTCCACATGT

TGCATTTCTTGATTTTCCACAAGCAGTTAGGCCCAGCCCAATAGTGATAAACATGTGTTCGTCCACTTTTCGAGGTGGTG

GGTTCCAAAATGGGCCAGTTACAAGCCCAGTTATATTAGTGAAAAATTTATGGGCTGTTGGTGTGTCATTAAAGGCAGGT

AGAATTGGCATTATGGGAGTTGATGGTAGTAATGCATCTTCATACACTATAATTCCTCTAGTTGTGGTGTTGTCAAATGG

CACCCCGGCTGCACTAGCATAGGGGTTAGCCGTCATGTAGTACGACGCCGGTAGTTGATCGGCCGTCAAGAGGACGTCAG

TCGTCTGGCCCGGCCCGGTGACAACTACATCCGTGACGTAGGGATCAGTGTAAGCAGCATCAACCGCGACAACTTTCATT

TTATGATTGGCGATCTTGAAAAAAAGTTGGGTTATTGAGTGCAGCGTTGATGATACGTAGGAGATATGTTTTCGTGTTCA

CTTTCACTTGTACTGTTCCTAATACAAATGAAAACGACATCAGCTAGCAGACAGCAAGTCCAAGAGATTTTCACCTCTGT

TG

>Laccase5 (Reverse primer)

CCAGAGATCATGTGCAATGACCAGGTGAATTTGGCCTATCGACTGCAGGGTTATAATTGCCAAAGCCTTGTGCCAATACA

TGGAAATTGAATCCATGCAAGTGAATAGGATGATTTTCTATCCCAATCAAAGCAGTATTTTGGAACACAATTTCAACTGT

GGAATTGAACTTAATTTTCTTCACTTTTGTCGATTTTTTCGTCATTATAATAGCAGGATTCATGCTATTATTTGGGTTTG

TATAGTCAAACTTCAATGGTGGTTTGTCTGGGAAATCAGTAGTGTAAATTCCACCAACATTGTAGAAAAAGGCTTCCAAC

ATTGAAATCTTGTCTGGAAACTGGAAAGATGCATTGTTCATACTAGCCGAAAATCGTTGTCCATTTGGGCCTCCACATGT

TGCATTTCTTGATTTTCCACAAGCAGTTAGGCCCAGCCCAATAGTGATAAACATGTGTTCGTCCACTTTTCGAGGTGGTG

GGTTCCAAAATGGGCCAGTTACAAGCCCAGTTATATTAGTGAAAAATTTATGGGCTGTTGGTGTGTCATTAAAGGCAGGT

AGAATTGGCATTATGGGAGTTGATGGTAGTAATGCATCTTCATACACTATAATTCCTCTAGTTGTGGTGTTGTCAAATGG

CACCCCGGCTGCACTAGCATAGGGGTTAGCCGTCATGTAGTACGACGCCGGTAGTTGATCGGCCGTCAAGAGGACGTCAG

TCGTCTGGCCCGGCCCGGTGACAACTACATCCGTGACGTAGGGATCAGTGTAAGCAGCATCAACCGCGACAACTTTCATT

TTATGATTGGCGATCTTGAAAAAAAGTTGGGTTATTGAGTGCAGCGTTGATGATACGTAGGAGATATGTTTTCGTGTTCA

CTTTCACTTGTACTGTTCCTAATACAAATGAAAACGACATCAGCTAGCAGACAGCAAGTCCAAGAGATTTTCACCTCTGT

TG

**2. miR398: TATGTTCTCAGGTCGCCCCTG**

| miRNA_Acc. | miRBase ID | Mature_miRNA | Target_Acc. | Target_Desc. | Expectation | UPE | miRNA_start | miRNA_end | Target_start | Target_end | miRNA_aligned_fragment (5’-3’) | Target_aligned_fragment (5’-3’) | Inhibition |
| --- | --- | --- | --- | --- | --- | --- | --- | --- | --- | --- | --- | --- | --- |
| stu-known-mir61 | mir398 | TTGTGTTCTCAGGTCACCCCT | PGSC0003DMT400026824 | Serine-threonine protein kinase, plant-type | 4 | 16.926 | 1 | 21 | 3655 | 3675 | UUGUGUUCUCAGGUCACCCCU | ACUGGUGACUUGAGCAUACGA | Cleavage |

> PGSC0003DMT400026824_Serine-threonine protein kinase, plant-type 5’- ACUGGUGACUUGAGCAUACGA -3’

: . . : : : : : : . . : : : . : . : : . :

>miR398 3’ -UCC CCA CUGGACUCUUGUGUU - 5’

>Serine-threonine protein kinase1(F)

TGTGTTTTTTTAATTCATATGGCTGTCGCGGTTGTGCCTCTCATATTTTATTTGATTTAAAGCCGGAACGGATTACGATG

TTGATTCTTTCTCTGCAGATACATGCTTAATTCGAGTTTACCCGAAAGCTTGGGGAGCCTTCCGTGATCTCATAGAGTTC

AATTTTTCATCCAATTTATTGCATGGCAAAATTCCTATTGAGATTGGAAATTTAAAGGCTGCAACACTCATTGATCTGTC

AAAAAATGATTTTTTTGGTATGATCCCTAGCACTCTAGAGGGTCTGGATAGATTGATTAGTCTTTCTCTAGCACATAATA

AATTAGATGGGCCTATTCCAGATTCATTTAGAAAAATGCTGGCCTTGGAATACTTGGATTTGTCCAGTAACAATCTTAGT

GGTGAAATCCCAAAGTCATTAGAAGCTCTTGTGTATCTCAAATACTTGAACTTCTCATTTAATGAATTCAGTGGAGAAAT

TCCCACGGGTGGTCCTTTTGCAAATGCCACAGATCAATCTTTCTTGTCCAATTATGGGCTCTGTGGTGATTCTAAGTTTC

ATGTTTCACCATGTGTCATCAAATCTCCCAAGAGGTCAAAGAAAAAAAAGGCAATATTGGTTTTGTACATCCTTTTGGGA

GTAGGTATGCTATTTCTTGCATTAGCCGTCACATATGTATTTTTGAGATTGAGAAAGAAAAAAAAGAATGCAGGTCAAGC

TGATGTGTCTCTGATAAAATGTCATGAAAGAATTTCCTATTATGAACTTGAACAAGCAACCGAAGGATTCAGCGAAAGCA

ACTTGCTTGGTAACGGGAGTTTCAGCAAGGTCTACAAAGGGATACTTAAAGGATGGTACTCTTTTGGGCATCAAGGGTAT

TCATGTTGCAATTTGGGAGGGGTGCATTCAAAAGATTTGATACAGCATGTGAGATGTGCGCATGTCGGTCCATTACCGTC

AGGCC

>2Serine-threonine protein kinase2(R)

CGTATAACTTTTGATGCACCCTCCATTGCACATTGAATACCTTTGCTGCCAAAAGAGTACCATCCTTAAGTATCCCTTTG

TAGACCTTGCTGAAACTCCCGTTACCAAGCAAGTTGCTTTCGCTGAATCCTTCGGTTGCTTGTTCAAGTTCATAATAGGA

AATTCTTTCATGACATTTTATCAGAGACACATCAGCTTGACCTGCATTCTTTTTTTTCTTTCTCAATCTCAAAAATACAT

ATGTGACGACTAATGCAAGAAATAGCATACCTACTCCCAAAAGGATGTACAAAACCAATATTGCCTTTTTTTTCTTTGAC

CTCTTGGGAGATTTGATGACACATGGTGAAACATGAAACTTAGAATCACCACAGAGCCCATAATTGGACAAGAAAGATTG

ATCTGTGGCATTTGCAAAAGGACCACCCGTGGGAATTTCTCCACTGAATTCATTAAATGAGAAGTTCAAGTATTTGAGAT

ACACAAGAGCTTCTAATGACTTTGGGATTTCACCACTAAGATTGTTACTGGACAAATCCAAGTATTCCAAGGCCAGCATT

TTTCTAAATGAATCTGGAATAGGCCCATCTAATTTATTATGTGCTAGAGAAAGACTAATCAATCTATCCAGACCCTCTAG

AGTGCTAGGGATCATACCAAAAAAATCATTTTTTGACAGATCAATGAGTGTTGCAGACTTTAAATTTCCAATCTCAATAG

GAATTTTGCCATGCAATAAATTGGATGAAAAATTGAACTCTATGAGATCAGGAAGGCTCCCCAAGCTTTCGGGTAAACTC

GAATTAAGCATGTTATCTGCTAGAATAAAAGAATATCTCATACTGGTAACTTTTCCTAAGCAGTGGGGCCCCCGCGACCA

GAA

>3Serine-threonine protein kinase3 (F)

GGATCGTTGAGATATCTTTCTCTAGCTGATAACATGCTTAATTCGAGTTTACCCGAAAGCTTGGGGAGCCTTCCTGATCT

CATAGAGTTCAATTTTTCATCCAATTTATTGCATGGCAAAATTCCTATTGAGATTGGAAATTTAAAGGCTGCAACACTCA

TTGATCTGTCAAAAAATGATTTTTTTGGTATGATCCCTAGCACTCTAGAGGGTCTGGATAGATTGATTAGTCTTTCTCTA

GCACATAATAAATTAGATGGGCCTATTCCAGATTCATTTAGAAAAATGCTGGCCTTGGAATACTTGGATTTGTCCAGTAA

CAATCTTAGTGGTGAAATCCCAAAGTCATTAGAAGCTCTTGTGTATCTCAAATACTTGAACTTCTCATTTAATGAATTCA

GTGGAGAAATTCCCACGGGTGGTCCTTTTGCAAATGCCACAGATCAATCTTTCTTGTCCAATTATGGGCTCTGTGGTGAT

TCTAAGTTTCATGTTTCACCATGCGTCATCAAATCTCCCAAGAGGTCAAAGAAAAAAAAGGCAATATTGGTTTTGTACAT

CCTTTTGGGAGTAGGTATGCTATTTCTTGCATTAGCCGTCACATATGTATTTTTGAGATTGAGAAAGAAAAAAAAGAATG

CAGGTCAAGCTGATGTGTCTCTGATAAAATGTCATGAAAGAATTTCCTATTATGAACTTGAACAAGCAACCGAAGGATTC

AGTGAAAGCAACTTGCTTGGTAACGGGAGTTTCAGCAAGGTCTACAAAGGGATACTTAAGGGATGGTACTCTTTTGGCAT

CAAAGGTATTCAATGTGCATTGGAGGGTGCATTCAGAAGTTTTGATACAGAATGTGAGATGTACGCCAGTTGCGGCCCAC

CCCCTCGCGCGGGGCAAAAAGG

>4Serine-threonine protein kinase4 (R)

CTTCACTTTTGATGCACCCTCCAATTGCACATTGAATACCTTTGATGCCAAAAGAGTACCATCCTTAAGTATCCCTTTGT

AGACCTTGCTGAAACTCCCGTTACCAAGCAAGTTGCTTTCACTGAATCCTTCGGTTGCTTGTTCAAGTTCATAATAGGAA

ATTCTTTCATGACATTTTATCAGAGACACATCAGCTTGACCTGCATTCTTTTTTTTCTTTCTCAATCTCAAAAATACATA

TGTGACGGCTAATGCAAGAAATAGCATACCTACTCCCAAAAGGATGTACAAAACCAATATTGCCTTTTTTTTCTTTGACC

TCTTGGGAGATTTGATGACGCATGGTGAAACATGAAACTTAGAATCACCACAGAGCCCATAATTGGACAAGAAAGATTGA

TCTGTGGCATTTGCAAAAGGACCACCCGTGGGAATTTCTCCACTGAATTCATTAAATGAGAAGTTCAAGTATTTGAGATA

CACAAGAGCTTCTAATGACTTTGGGATTTCACCACTAAGATTGTTACTGGACAAATCCAAGTATTCCAAGGCCAGCATTT

TTCTAAATGAATCTGGAATAGGCCCATCTAATTTATTATGTGCTAGAGAAAGACTAATCAATCTATCCAGACCCTCTAGA

GTGCTAGGGATCATACCAAAAAAATCATTTTTTGACAGATCAATGAGTGTTGCAGACTTTAAATTTCCAATCTCAATAGG

AATTTTGCCATGCAATAAATTGGATGAAAAATTGAACTCTATGAGATCAGGAAGGCTCCCCAAGCTTTCGGGTAAACTCG

AATTAAGCATGTTATCAGCTAGAGAAAGATATCTCATACTGGTAACTTTTCCTAAGCAAGGGGGCCCGCGGACAGAG

>5Serine-threonine protein kinase5 (F)

GGGCGTCCGTGATATCGGTCCCTAGCTGAGAACATGCTTAATTCGAGTTTACCCGAAAGCTTGGGGAGCCTTCCTGATCT

CATAGAGTTCAATTTTTCATCCAATTTATTGCATGGCAAAATTCCTATTGAGATTGGAAATTTAAAGGCTGCAACACTCA

TTGATCTGTCAAAAAATGATTTTTTTGGTATGATCCCTAGCACTCTAGAGGGTCTGGATAGATTGATTAGTCTTTCTCTA

GCACATAATAAATTAGATGGGCCTATTCCAGATTCATTTAGAAAAATGCTGGCCTTGGAATACTTGGATTTGTCCAGTAA

CAATCTTAGTGGTGAAATCCCAAAGTCATTAGAAGCTCTTGTGTATCTCAAATACTTGAACTTCTCATTTAATGAATTCA

GTGGAGAAATTCCCACGGGTGGTCCTTTTGCAAATGCCACAGATCAATCTTTCTTGTCCAATTATGGGCTCTGTGGTGAT

TCTAAGTTTCATGTTTCACCATGCGTCATCAAATCTCCCAAGAGGTCAAAGAAAAAAAAGGCAATATTGGTTTTGTACAT

CCTTTTGGGAGTAGGTATGCTATTTCTTGCATTAGCCGTCACATATGTATTTTTGAGATTGAGAAAGAAAAAAAAGAATG

CAGGTCAAGCTGATGTGTCTCTGATAAAATGTCATGAAAGAATTTCCTATTATGAACTTGAACAAGCAACCGAAGGATTC

AGTGAAAGCAACTTGCTTGGTAACGGGAGTTTCAGCAAGGTCTACAAAGGGATACTTAAGGGATGGTACTCTTTTGGCAT

CAAAGGTATTCAATGTGCATTGGAGGGTGCATTCAGAAGTTTTGATACAGAATGTGAGATGTACGCCAGTTGCGGCCCAC

CCCCTCGCGCGGGGCAAAAAGG
